# Atypical cadherin CELSR2 acts as a therapeutic target for glioma through WNT3A/β-catenin signaling

**DOI:** 10.1038/s41419-025-08116-8

**Published:** 2025-11-03

**Authors:** Aimei Liu, Xin Geng, Xinyue Li, Yue Xi, Qing Han, Xiangyu Wang, Yajing Shen, Libing Zhou

**Affiliations:** 1 School of Life Sciences and Health, University of Health and Rehabilitation Sciences, Qingdao, Shandong P. R. China; 2 Department of Pain, University of Health and Rehabilitation Sciences Qingdao Hospital (Qingdao Municipal Hospital), Qingdao, Shandong P. R. China; 3 Department of Neurosurgery, The First Affiliated Hospital of Jinan University, Guangzhou, P. R. China; 4 Guangdong-Hongkong-Macau CNS Regeneration Institute of Jinan University, Key Laboratory of CNS Regeneration (Ministry of Education), Guangdong Key Laboratory of Non-human Primate Research, Guangzhou, P. R. China; 5 Co-innovation Center of Neuroregeneration, Nantong University, Nantong, Jiangsu P. R. China; 6 Center for Exercise and Brain Science, School of Psychology, Shanghai University of Sport, Shanghai, P.R. China

**Keywords:** CNS cancer, Cancer therapy

## Abstract

Glioma is the most common primary brain tumors and has a high recurrence and mortality rate after surgery. Most gliomas are of astrocytic origin. We recently demonstrated that Celsr2 is essential for injury-induced responses and functions of astrocytes, while its role in the development and treatment of gliomas remains unexplored. In this study, an increase of CELSR2 expression was identified in patient glioma samples and glioma cell lines, and higher levels of CELSR2 correlate with poorer patient survival as indicated by TCGA data. In cultured glioma cells, *CELSR2* knockdown reduced proliferation and caused cell cycle arrest, which was further supported by proteomic analysis. *CELSR2* knockdown inhibited Wnt/β-catenin signaling, and the effect could be reversed by activating β-catenin using GSK-3β inhibitor in glioma cells. WNT3A efficiently enhanced the proliferation of glioma cells and activated the downstream signaling, which were significantly compromised by *CELSR2* knockdown. We developed magnetic nanoparticles loaded with *CELSR2*-siRNA, which suppressed tumor growth in glioma-inoculated nude mice. In conclusion, CELSR2 positively regulates glioma development through WNT3A/β-catenin signaling and inhibiting CESLR2 is a novel therapeutic strategy for gliomas.

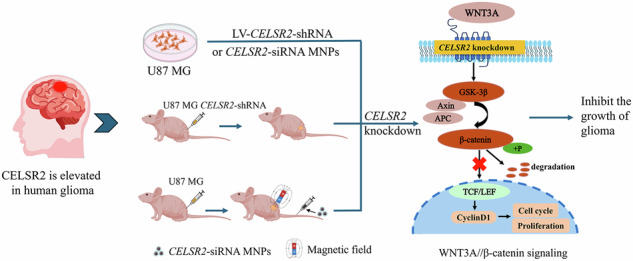

## Introduction

Gliomas are the most common primary brain tumor originating from glial cells. Their high invasiveness, difficulty in complete resection, frequent recurrence and poor prognosis severely diminish the life quality of patients and cause heavy burden on patients, their families, and society. Gliomas account for ~23% of all primary brain tumors and 81% of malignant brain tumors and are responsible for most deaths from primary brain tumors [1]. Traditional therapeutic approaches, such as surgery, chemotherapy, and radiation therapy, have shown limited improvement in the prognosis of glioma patients. Over the years, the molecular mechanism of the occurrence and development of glioma has been gradually revealed, and innovative therapeutic strategies have been proposed, such as targeted therapy [2, 3], immunotherapy [4, 5]. However, the prognosis of some glioma patients is still very poor, for instance, the median survival time for glioblastoma patients is less than two years after effective standard therapies [6]. Gliomas are composed of a heterogeneous group of tumors diagnosed according to their histological characteristics and different molecular biomarkers, as defined in the fifth edition of the WHO Classification of Tumors of the Central Nervous System (WHO CNS5) [7]. Some molecular markers have been identified for classification, typing, grading, prognosis and therapeutic management of gliomas [8, 9]. Specific biomarkers are important for the diagnosis of gliomas and it is urgently needed to actively seek new targets for glioma individualized treatment.

Gliomas were classified as grade 1–4 from low to high malignancy in the WHO CNS4 [10], and divided into 5 different families according to histological and molecular pathological features in WHO CNS5 [7, 11]. Adult-type diffuse gliomas include three tumor types: oligodendroglioma of isocitrate dehydrogenase (IDH) mutant and 1p/19q codeleted showing the best prognosis, astrocytoma of IDH mutant with intermediate prognosis, and glioblastoma of IDH wild-type with poor prognosis. Pediatric-type diffuse gliomas contain low-grade and high-grade tumors with aggressive behavior, poor prognosis in which of histone 3k27 altered in children. Circumscribed astrocytic gliomas are characterized by growth restriction and frequent BRAF alterations, with a favorable prognosis. Ependymomas’ prognosis is related to their molecular biomarkers and location. While surgery, radiotherapy, and chemotherapy are the primary treatments for glioma, personalized treatment strategies based on the tumor-intrinsic dominant signaling pathways have improved the prognosis for some patients [11]. Therefore, there is an urgent need to explore the tumor-intrinsic dominant signaling pathways with targeted properties in order to provide effective personalized treatment.

Wnts are composed of a family of secreted glycoproteins and the Wnt signaling has been identified to be one of the key cascades involved in tumorigenesis through the canonical pathway (β-catenin dependent) [12–14]. In the Wnt/β-catenin pathway, binding of Wnts to their receptors activates the recruitment of a destruction complex to the cell membrane and separates β-catenin from this complex, which subsequently prevents β-catenin from phosphorylation and degradation. Cytoplasmic accumulated β-catenin is then translocated to the nucleus, where it activates target gene transcription [15]. Mutated Wnt signaling pathway components could lead to a variety of growth-related pathological changes and the occurrence of cancer [16]. The Wnt/β-catenin pathway is highly activated in glioma [17]. Abnormally activated Wnt/β-catenin pathway significantly promotes the pathogenesis and progression of glioma, playing a key role in glioma cell proliferation, migration, invasion, and angiogenesis [17, 18]. CELSR1-3 plays an important role in the epithelium and nervous system via the WNT signaling pathway [19, 20], but the effect of CELSR2 on glioma growth through the regulation of the WNT pathway has not been reported in the relevant literature.

*CELSR2* gene encodes an atypical cadherin receptor with a seven-transmembrane motif. As one of the core members of planar tissue polarity genes, *CELSR2* is involved in multiple biological processes of neurodevelopment [21]. Studies have shown that the expression of CELSR2 is significantly increased in hepatocellular carcinoma and can be used as a new prognostic biomarker for hepatocellular carcinoma [22]. In addition, CELSR2 regulates Schwann cell migration and proliferation through the Wnt/β-catenin pathway [23]. Our previous studies have shown that CELSR2 is widely expressed in astrocytes, oligodendrocytes and activated microglia [24, 25]. Since most gliomas originate from glial cells, we hypothesized that CELSR2 might be involved in the development and treatment of glioma.

To address our hypothesis, we first analyzed the online TCGA data related to glioma patients to reveal CELSR2 expression in glioma tissues and its correlation with patient overall survival (OS). CELSR2 expression was further evaluated in glioma tissues from clinical patients and glioma cell lines. In cultured glioma cells, *CELSR2* was knocked down by *CELSR2*-shRNA and the effects on glioma growth were investigated both in vitro and in vivo. The potential molecular mechanisms were elucidated by analyzing *CELSR2* knockdown-induced changes of proteomic profiles and Wnt/β-catenin pathways. Finally, we developed nanoparticles loaded with *CELSR2*-siRNA and evaluated their therapeutic effects on glioma growth in nude mice after xenografting. Our data demonstrate that CELSR2 promotes glioma growth through WNT3A/β-catenin signaling and inhibiting CELSR2 is a novel strategy for glioma therapy.

## Results

### CELSR2 expression is elevated in glioma tissues from clinical patients and glioma cell lines

**Fig. 1:**
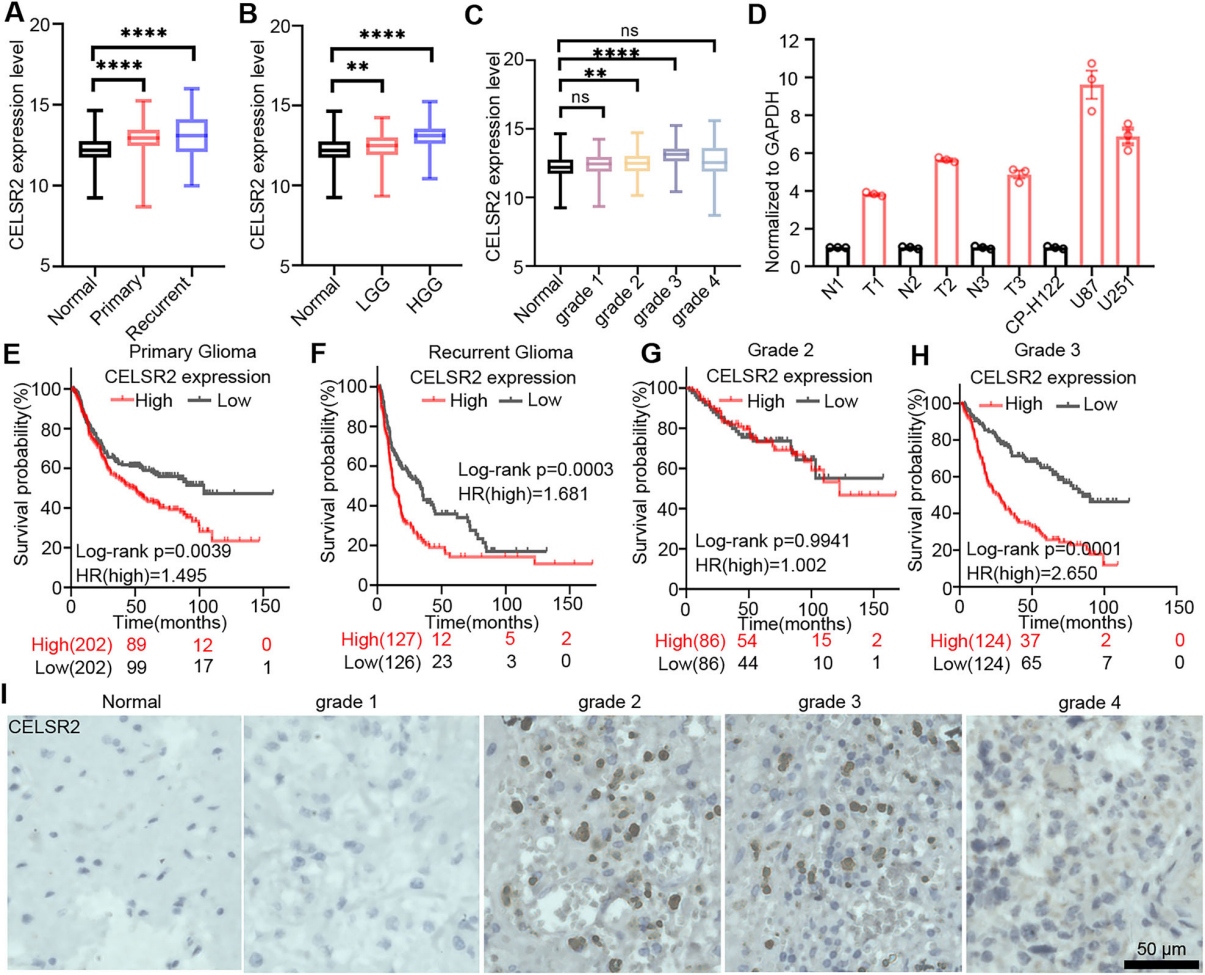
CELSR2 expression is elevated in glioma tissues. **A** Analysis of TCGA data reveals significantly elevated *CELSR2* mRNA levels in primary and recurrent glioma tissues compared to normal tissues (*n* = 1081 for normal; *n* = 662 for primary; *n* = 277 for recurrent). **B** Analysis of TCGA data demonstrates a significant upregulation of CELSR2 mRNA in low-grade glioma (LGG) and high-grade glioma (HGG) tissues relative to normal brain tissues (*n* = 1081 for normal; *n* = 423 for LGG; n = 502 for HGG). **C** Analysis of TCGA data indicates that *CELSR2* mRNA expression is significantly increased in Grade 2 and Grade 3 gliomas compared to normal brain tissues (*n* = 1081 for normal; *n* = 115 for Grade 1; *n* = 289 for Grade 2; *n* = 254 for Grade 3; *n* = 248 for Grade 4). **D** RT-qPCR analysis shows elevated *CELSR2* expression in clinical glioma tissues and glioma cell lines (*n* = 3). N, para-tumoral normal tissue; T, tumor; T1: Grade 2; T2, T3: Grade 3; CP-H122, normal astrocyte line. **E** Kaplan–Meier survival curves illustrate reduced overall survival in patients with primary glioma (all WHO grades combined, *n* = 202). **F** Kaplan–Meier survival curves demonstrate decreased overall survival in patients with recurrent glioma (all WHO grades combined, *n* = 127 or 126). **G** Kaplan–Meier survival analysis reveals poorer prognosis in Grade 2 glioma patients (all WHO grades combined, *n* = 86). **H** Kaplan–Meier survival curve of grade 3 glioma patients (All WHO grade survival, *n* = 124). **I** Immunohistochemical staining showing CELSR2 levels in Grade 1-Grade 4 glioma tissues (*n* = 3). Scale bar is 50 μm. Data are represented as mean ± SEM. **P < 0.01, ****P < 0.0001, one-way ANOVA analysis of variance with Tukey’s multiple comparison in (**A**–**C**).

Our previous study has shown that Celsr2 is expressed in astrocytes, oligodendrocytes and activated microglia in the central nervous system (CNS) and regulates reactive astrocytes involved in neural repair [25]. To test whether CELSR2 is also involved in the development of glioma, we first studied the expression of CELSR2 in the glioma tissues from the clinical patients. Analysis of TCGA and GTEx data obatained from the online database UCSC Xena (http://xena.ucsc.edu) demonstrated that CELSR2 mRNA levels were significantly upregulated in both primary and recurrent gliomas compared to normal brain tissue (Fig. 1A). Consistently, CELSR2 mRNA levels were markedly elevated in low-grade gliomas (Grade 1 and Grade 2) and high-grade gliomas (Grade 3 and Grade 4) relative to normal brain tissue (Fig. 1B). Notably, CELSR2 mRNA expression was significantly higher in Grade 2, Grade 3 and recurrent Grade 4 gliomas when compared to the normal control group (Fig. 1C, Supplementary Fig. 1A). To confirm this finding, we collected glioma and para-tumor tissues from clinical patients (Grade 2 and Grade 3 gliomas) and studied CELSR2 expression using RT-qPCR. The expression level of *CELSR2* mRNA was significantly higher in glioma tissues than that in para-tumor tissues (Fig. 1D). In patients with primary and recurrent glioma, the survival probability was negatively correlated with CELSR2 expression in the glioma tissues: patients with higher *CELSR2* mRNA levels in the glioma tissues showed a shorter survival time (Fig. 1E, F). Meanwhile, although CELSR2 expression was significantly upregulated in Grade 2, Grade 3 and recurrent Grade 4 gliomas, a significantly shorter survival time was only observed in patients with elevated CELSR2 mRNA levels specifically in Grade 3 gliomas and recurrent Grade 4 gliomas (Fig. 1G, H; Supplementary Fig. 1B–D). Immunohistochemical staining showed an increased expression of CELSR2 proteins in Grade 2 and Grade 3 glioma tissues (Fig. 1I). Similarly, *CELSR2* mRNA was significantly upregulated in two glioma cell lines (U87 MG and U251) compared to the normal astrocyte line (CP-H122) (Fig. 1D). The results suggest that CELSR2 is associated with the development of glioma, and inhibiting CELSR2 may be a potential strategy for glioma treatment.

### *CELSR2* knockdown (KD) reduces proliferation of glioma cells and disturbs the cell cycle

**Fig. 2:**
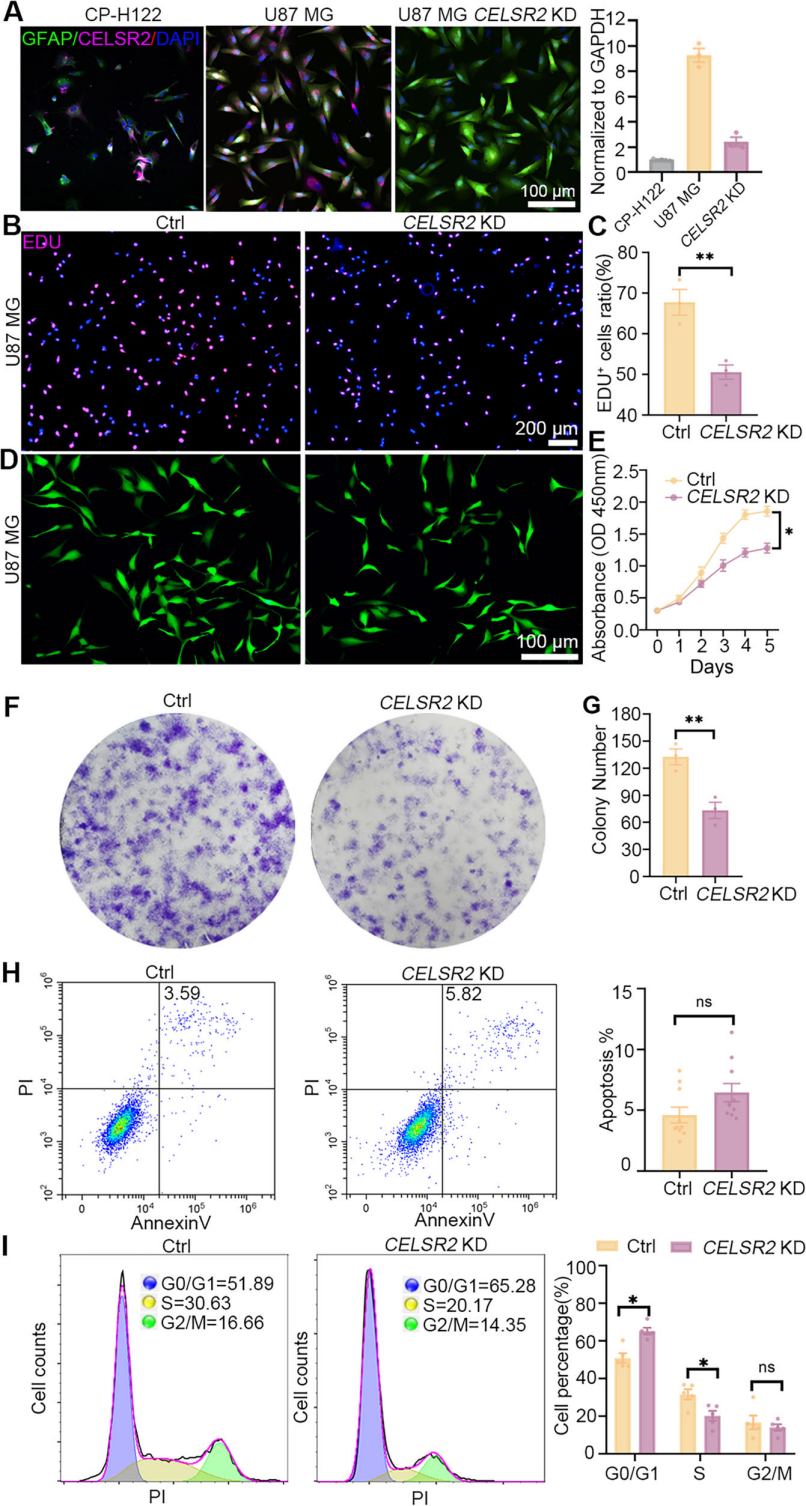
*CELSR2* KD inhibits glioma cell proliferation and disturbs cell cycle. **A** Anti-GFAP (green) and anti-CELSR2 immunofluorescence staining showed the expression of CELSR2 in CP-H122 cells, U87 MG cells transfected with the vector (pLVX-shRNA1-ZsGreen-puro, control) or *CELSR2*-shRNA (*CELSR2* KD), and the levels of *CELSR2* mRNA were further assessed by RT-qPCR, showing a downregulation in U87 MG cells transfected with *CELSR2*-shRNA. **B**–**E** EDU staining (**B**, **C**) and CCK-8 assay (**D**, **E**) showed a significant decrease in the number of proliferating cells in the CELSR2-KD group. **F**, **G** Two-dimensional culture assay showed reduced colonies in the CELSR2-KD group. **H** Using annexin V and propidium iodide (PI) staining, FACS analysis identified a comparable percentage of apoptotic cells in the CELSR2-KD group and the control group. **I** FACS analysis of PI-stained cells showed a significant increase in the G0/G1 ratio and a significant decrease in the S phase in the CELSR2-KD group. Scale bar is 100 μm in (**A**, **D**) and 200 μm in (**B**). Data are represented as mean ± SEM. *P < 0.05, **P < 0.01, Two-way repeated measures ANOVA with Bonferroni’s post hoc correction in (**E**) and Student’s *t*-test in others, *n* = 3 in (**A**–**F**); *n* = 10 in (**H**); *n* = 5 in (**I**).

To clarify the role of CELSR2, we knocked down *CELSR2* in the glioma cell line (U87 MG) and primary glioma cells (Grade 3, Supplementary Fig. 2A) using lentiviral vectors expressing *CELSR2*-shRNA. Immunostaining showed that CELSR2 was positive in GFAP-positive astrocytes of both CP-H122 and U87 MG cell lines, but a rare detectable CELSR2-positive signal was found in the *CELSR2*-shRNA treated U87 MG cell lines and *CELSR2*-shRNA treated Grade 3 primary glioma cells (Fig. 2A, Supplementary Fig. 2B). In *CELSR2*-shRNA-transfected U87 MG cells and *CELSR2*-shRNA-transfected Grade 3 primary glioma cells (*CELSR2* KD), the expression of *CELSR2* mRNA was significantly downregulated compared to the vector (pLVX-shRNA1-ZsGreen-puro)-transfected cells (control), but still higher than that in normal astrocytes (CP-H122) (Fig. 2A, Supplementary Fig. 2C). We studied cell proliferation using EDU labeling and found a significant decrease in EDU-positive cells in the CELSR2-KD group compared to the control group (Fig. 2B, C; Supplementary Fig. 2D, E). A similar result of *CELSR2* KD inhibiting U87 MG cell proliferation was confirmed by CCK-8 assay (Fig. 2D, E). Using 2-dimensional culturing, we found that the colony number was significantly reduced in the CELSR2-KD group compared to the control group (Fig. 2F, G), indicating that *CELSR2* KD prevents U87 MG cells from forming colonies. To investigate whether *CELSR2* KD influences cell apoptosis, cultured cells were subjected to FACS analysis, showing no significant differences of PI and Annexin V double-positive population between the CELSR2-KD group and the control group (Fig. 2H; Supplementary Fig. 2F, G), suggesting that CELSR2 is not involved in apoptosis of U87 MG cells or Grade 3 primary glioma cells. We then studied the cell cycle distribution of U87 MG cells and Grade 3 primary glioma cells treated with *CELSR2*-shRNA. Strikingly, *CELSR2* KD led to a significant decrease in the S phase and a concurrent increase in the G0/G1 phase in U87 MG cells (Fig. 2I), and in Grade 3 primary glioma cells, *CELSR2* KD led to a significant decrease both in the S and G2/M phase and a concurrent increase in the G0/G1 phase (Supplementary Fig. 2H, I).

### *CELSR2* KD alters proteomic profiles and Wnt/β-catenin signaling in glioma cells

**Fig. 3:**
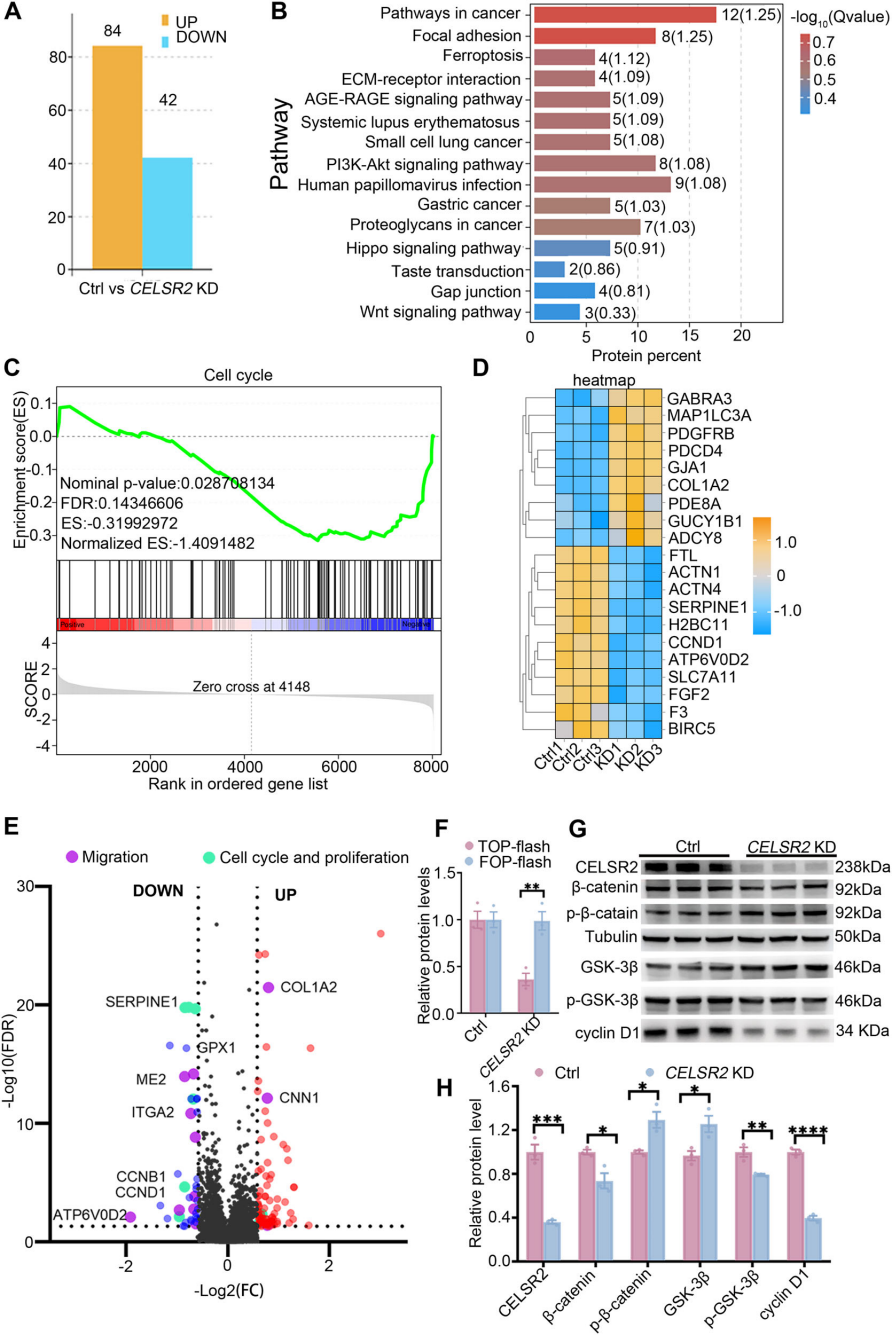
*CELSR2* KD alters proteomic profiles in glioma cells. **A**–**E** DIA proteomics of cultured U87 MG cells transfected without (control) or with *CELSR2*-shRNA (*CELSR2* KD) revealed 84 upregulated DEPs and 42 downregulated DEPs in the CELSR2-KD group compared to the control group (**A**; FDR < 0.05, |FoldChange| > 1.5), clustering in cancer pathways and the Wnt signaling pathway using KEGG pathway enrichment analysis (**B**). Downregulation of the cell cycle was shown by GSEA of DEPs (**C**; NES = −1.409, FDR = 0.143). The heatmap showed the top 20 DEPs (**D**), and the volcano diagram presented proteins with upregulation and downregulation in the CELSR2-KD group (**E**). **F** TOP/FOP flash luciferase assay showed a significant downregulation of Wnt/β-catenin signaling in the CELSR2-KD group. **G**, **H** Western blots of U87 MG cells showed the expression of CELSR2, β-catenin, GSK-3β, cyclin D1, p-β-catenin and p-GSK-3β in the CELSR2-KD group compared to the control group (**G**), with a significant upregulation of total GSK-3β, p-β-catenin and a significant downregulation of p-GSK-3β, total β-catenin and cyclin D1 (**H**). Data are represented as mean ± SEM. *P < 0.05, **P < 0.01, ***P < 0.001, ****P < 0.0001, triplicate experiments in each group, Student’s *t*-test.

To further explore potential molecular mechanisms of *CELSR2* KD-induced proliferation inhibition in glioma cells, we performed data-independent acquisition (DIA) proteomics with protein extracts from cultured U87 MG cells transfected without (control) or with *CELSR2-*shRNA (*CELSR2* KD). We identified 126 differentially expressed proteins (DEPs; FDR < 0.05, |FoldChange| >1.5) between the control and CELSR2-KD groups, including 84 DEPs with upregulation and 42 DEPs with downregulation (Fig. 3A). We performed KEGG pathway enrichment analysis of 126 DEPs and found that these DEPs were clustered in signaling pathways related to cancer and Wnt signaling pathway (Fig. 3B). Twelve DEPs were identified to be involved in pathways in cancer and three DEPs (CCND1, Fosl1, Frizzled) were related to the Wnt signaling pathway. Gene set enrichment analysis (GSEA) demonstrated a significant downregulation of cell cycle in the CELSR2-KD group compared to the control group (Fig. 3C; NES = −1.409, FDR = 0.143). The heatmap showed the relatively stable expression of 20 top DEPs involved in cell proliferation and cycle, such as cyclin D1(CCND1) in three batches of cultured cells (Fig. 3D). The downregulation of cell cycle and proliferation related proteins, such as CCND1, CCNB1 and SERPINE1, were identified in the volcano diagram of 126 DEPs (Fig. 3E). We detected the activation of Wnt/β-catenin signaling pathway through the TOP/FOP flash luciferase assay and found that *CELSR2* KD inhibited the transcriptional activity of T-cell factor/lymphoid enhancer-binding factor (TCF/LEF) in U87 MG cells (Fig. 3F). Western blots of U87 MG cell extracts revealed a significant upregulation of total GSK-3β and phosphorylated β-catenin(p-β-catenin) and a significant downregulation of phosphorylated GSK-3β(p-GSK-3β), total β-catenin and cyclin D1 in the CELSR2-KD group compared to the control group (Fig. 3G, H). Thus, CELSR2 regulates the proliferation and cell cycle of glioma cells possibly through the Wnt/β-catenin signaling pathway.

### Suppressing GSK-3β activation reverses proliferation inhibition induced by *CELSR2* KD in glioma cells

**Fig. 4:**
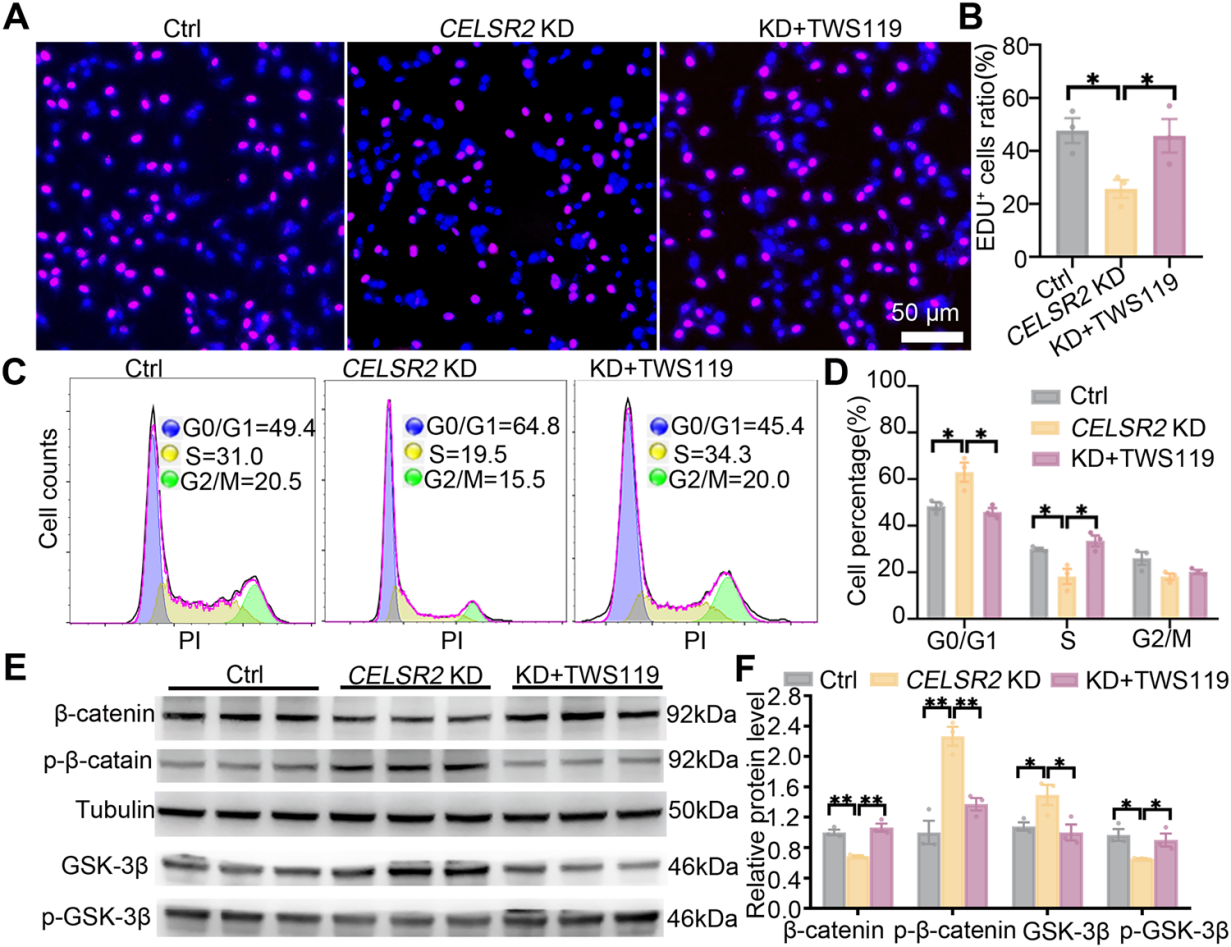
GSK-3β inhibitor TWS119 reverses *CELSR2* KD-induced proliferation inhibition in glioma cells. **A**, **B** Cultured U87 MG cells transfected with *CELSR2*-shRNA were treated without or with TWS119 (*CELSR2* KD or KD + TWS119), and cells without *CELSR2*-shRNA transfection were used as the control. EDU labeling showed a decrease in EDU^+^ cell density in the CELSR2-KD group compared to the control and the *CELSR2* KD + TWS119 group. **C**, **D** Flow cytometry of cultured cells revealed an increase in the G0/G1 phase and a decrease in the S phase in the CELSR2-KD group compared to the control and the *CELSR2* KD + TWS119 group. **E**, **F** Western blots showed lower expression of β-catenin and p-GSK-3β and higher expression of p-β-catenin and GSK-3β in the CELSR2-KD group compared to the control and the *CELSR2* KD + TWS119 group. Scale bar is 50 μm in (**A**). Data are represented as mean ± SEM. *P < 0.05; **P < 0.01; three independent experiments in each group; one-way ANOVA analysis of variance with Tukey’s multiple comparison.

In the Wnt/β-catenin signaling pathway, the suppression of GSK-3β is induced upon binding of Wnts to their receptors, which subsequently stabilizes β-catenin to translocate into the nucleus and activate TCF/LEF transcription factors [26]. To further confirm whether CELSR2 is involved in the regulation of Wnt/β-catenin signaling pathway to influence proliferation and cell cycle of glioma cells, we used the GSK-3β inhibitor TWS119 to activate Wnt/β-catenin. In the CELSR2-KD group, the ratio of EDU-positive cells was significantly increased after TWS119 treatment (Fig. 4A, B), and there was a significant increase in the S phase and a significant decrease in G0/G1 after TWS119 treatment (Fig. 4C, D). Meanwhile, the expression of GSK-3β and p-β-catenin was significantly decreased and the expression of p-GSK-3β and β-catenin was significantly increased in the CELSR2-KD group with TWS119 treatment compared to those without TWS119 treatment (Fig. 4E, F). Thus, *CELSR2* KD-induced proliferation reduction and cell cycle arrest of glioma cells could be reversed by activating the downstream signaling of the Wnt/β-catenin pathway.

### *CELSR2* KD compromises WNT3A-induced proliferation of glioma cells

**Fig. 5:**
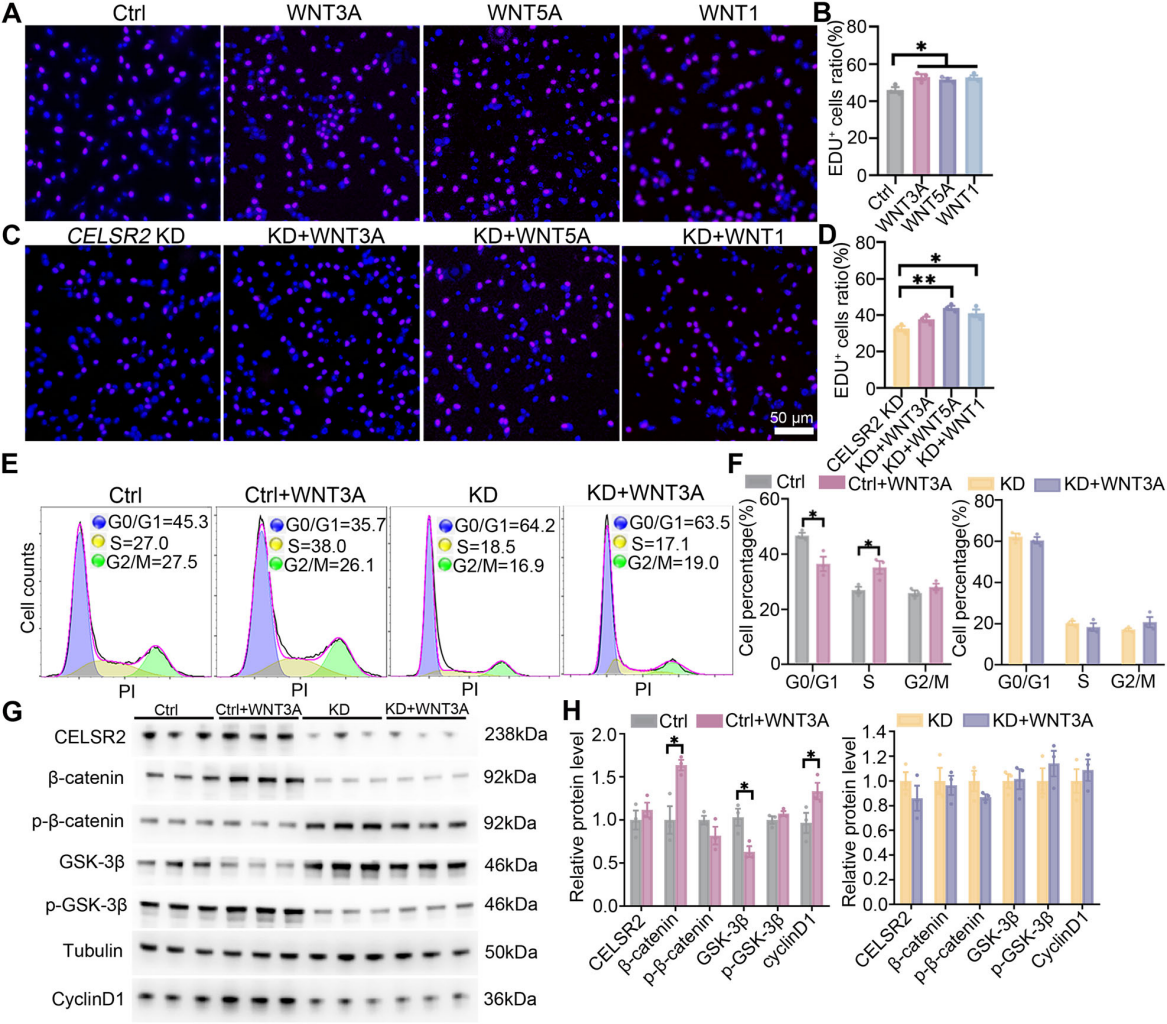
*CELSR2* KD compromises WNT3A-induced proliferation of U87 MG cells. **A**, **B** Administration of WNT3A, WNT5A and WNT1 significantly enhanced cell proliferation of cultured glioma cells, as revealed by EDU staining. **C**, **D** In CELSR2-KD U87 MG cells, WNT5A and WNT1, but not WNT3A, significantly enhanced cell proliferation as identified by EDU labeling. **E**, **F** Flow cytometry showed a decrease in the G0/G1 ratio and an increase in the S phase in the control+WNT3A group (U87 MG cells treated with WNT3A) compared to the control group (U87 MG cells), and no significant change in the KD group (CELSR2-KD U87 MG cells) compared to the KD + WNT3A group (CELSR2-KD U87 MG cells treated with WNT3A). **G**, **H** Western blots showed a significant increase of β-catenin and cyclin D1, and a significant decrease in GSK-3β in the Ctrl+WNT3A group compared to the control group, and there was no significant protein change in the KD group compared to the KD + WNT3A group. Scale bar is 50 μm in (**A**, **C**). Data are represented as mean ± SEM. *P < 0.05; **P < 0.01; ***P < 0.001; *n* = 3, Student’s *t*-test.

As ligands that activate the Wnt/β-catenin signaling pathway, WNTs are widely implicated in the development of glioma, and clinical data show that expression levels of WNT5A, WNT3A and WNT1 are closely correlated with the overall survival of patients with glioma [27–29]. To study whether CELSR2 serves as a potential receptor of WNT ligands to impact the development of glioma cells, WNT3A, WNT5A and WNT1 were administered to cultured U87 MG cells transfected without or with *CELSR2*-shRNA (*CELSR2* KD). EDU labeling revealed that the administration of WNT3A, WNT5A and WNT1 respectively enhanced the proliferation of cultured U87 MG cells (Fig. 5A, B). In cultured CELSR2-KD U87 MG cells, WNT5A and WNT1 administration still significantly increased the EdU-positive cell ratios compared to those cells without additional treatment, whereas WNT3A administration failed to enhance cell proliferation (Fig. 5C, D). The results suggest that *CELSR2* KD compromises WNT3A induced glioma proliferation. We further analyzed the effect of WNT3A on cell cycle using flow cytometry. In U87 MG cells (control), WNT3A treatment (control+WNT3A) significantly decreased the G0/G1 ratio and increased the S phase (Fig. 5E, F). But in CELSR2-KD U87 MG cells (KD), there was no significant change in cell cycle after WNT3A treatment (KD + WNT3A) (Fig. 5E, F). Meanwhile, we examined the expression levels of related proteins in the Wnt/β-catenin signaling pathway using Western blots in four different groups. There was a significant increase of β-catenin and cyclin D1 and a significant decrease of GSK-3β in the control+WNT3A group compared to the control group (Fig. 5G, H). There was no significant protein change in the CELSR2*-*KD group compared to the CELSR2-KD + WNT3A group (Fig. 5G, H). These results indicate that CELSR2 is a potential receptor of WNT3A in the Wnt/β-catenin signaling pathway and plays an important role in the proliferation and cell cycle regulation of glioma.

### *CELSR2*-KD U87 MG cells show slow growth in forming glioma in vivo

**Fig. 6:**
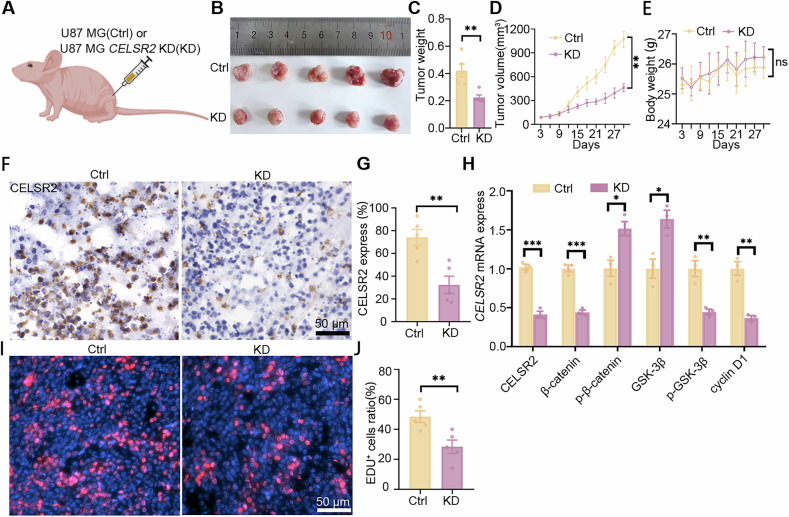
*CELSR2* KD U87 MG cells show slow growth in forming glioma in vivo. **A** Schematic diagram of U87 MG cell injection in nude mice. **B**–**E** Nude mice were inoculated with U87 MG cells transfected with or without *CELSR2*-shRNA (CELSR2-KD or control group). After one month of inoculation, tumor nodules appeared smaller (**B**) and their average weight was significantly lower (**C**) in the CELSR2-KD group compared to the control group. Dynamical measurement showed a significant decrease in tumor volume in the CELSR2-KD group compared to the control group (**D**), while animal body weight was comparable between the two groups (**E**). **F**, **G** Immunohistochemical staining of tumor tissues confirmed the downregulation of CELSR2 in the CELSR2-KD group. **H** In tumor nodules, there were a significant upregulation of p-β-catenin and GSK-3β and a significant downregulation of *CELSR2*, β-catenin, p-GSK-3β and cyclinD1 in the CELSR2-KD group compared to the control group identified by RT-qPCR. **I**, **J** EDU labeling revealed a significant decrease in proliferating tumor cells in the CELSR2-KD group compared to the control group. Scale bar is 50 μm in (**F**, **I**). Data are represented as mean ± SEM. Two-way repeated measures ANOVA with Bonferroni’s post hoc correction in (**D**, **E**) and Student’s *t*-test in others, *P < 0.05, **P < 0.01, ***P < 0.001, *n* = 5 in (**B**–**G**), (**I**) and (**J**), *n* = 3 in (**H**). A created with BioRender.

To further investigate the effect of inhibiting CELSR2 on glioma growth in vivo, we subcutaneously inoculated U87 MG cells with or without *CELSR2*-shRNA transfection (CELSR2-KD group or control group) in nude mice (Fig. 6A). After one month of inoculation, tumor tissues were collected, revealing a significant decrease in tumor volume and weight in the CELSR2-KD group compared to the control group (Fig. 6B, C). Dynamic measurement of tumor volume in live animals showed a significant difference between the two groups (Fig. 6D), while mouse body weight was comparable at the same time points in both groups (Fig. 6E). In the one-month growing tumor tissues, immunohistochemical staining revealed a significant downregulation of CELSR2 in the CELSR2-KD group compared to the control group (Fig. 6F, G). Additionally, we found a significant upregulation of p-β-catenin and GSK-3β, and a significant downregulation of *CELSR2*, β-catenin, p-GSK-3β and cyclinD1 in the CELSR2-KD group compared to the control group in the tumor nodule, as assessed by RT-qPCR (Fig. 6H). EDU labeling showed a significant decrease in tumor cell proliferation in the glioma (Fig. 6I, J). These results indicate that *CELSR2* KD could significantly inhibit the growth of established tumor nodule in vivo.

We further evaluated the impact of CELSR2 inhibition on glioma growth in the brain microenvironment using an orthotopic glioma model. U87 MG-Luciferase cells with or without CELSR2-shRNA transfection were orthotopically implanted into the brains of nude mice (CELSR2-KD group and control group, respectively; Supplementary Fig. 3A). After one month of post-implantation, in vivo bioluminescence imaging demonstrated that luciferase activity was significantly reduced in the CELSR2-KD group compared to the control group (Supplementary Fig. 3B, E). Additionally, we performed intratumoral injection of LV-shCELSR2 one week after the implantation of U87 MG-Luciferase cells. Bioluminescence imaging one month later also showed a significant decrease in tumor signal in the LV-shCELSR2 group relative to the control group (Supplementary Fig. 3C–E).

### Glioma growth is inhibited by administration of MNPs-loaded *CELSR2*-siRNA

**Fig. 7:**
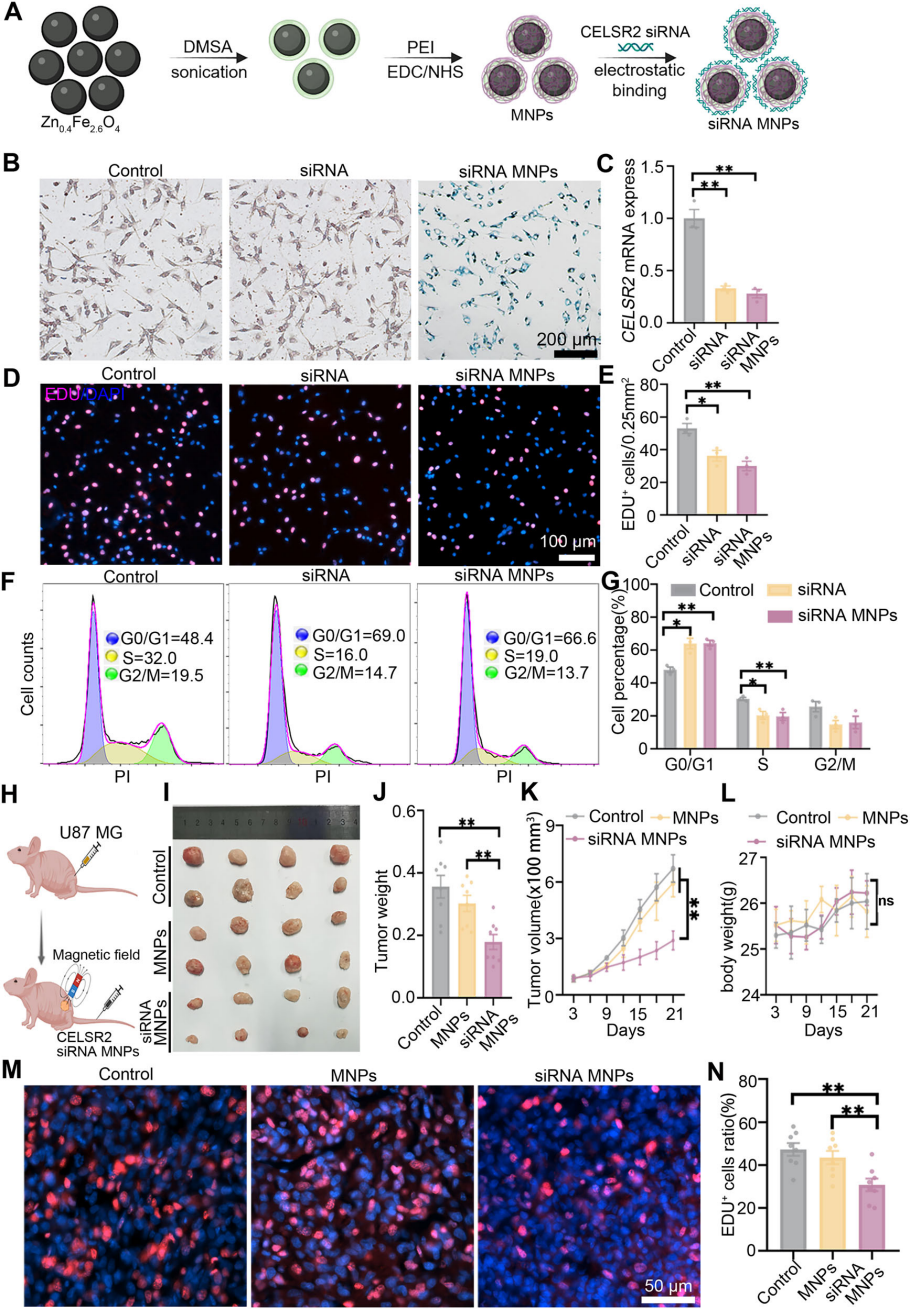
Glioma growth is inhibited by administration of MNPs-loaded *CELSR2*-siRNA. **A** Schematic diagram of MNPs-loaded *CELSR2*-siRNA. **B** PB stain revealed MNPs-loaded *CELSR2*-siRNA were ingested by cultured U87 MG cells. (**C**) *CELSR2* mRNA levels were significantly decreased in the siRNA group and siRNA MNPs group compared to the control group. **D**, **E** EDU labeling showed a significant decrease in proliferating cells in the siRNA group and siRNA MNPs group. **F**, **G** Flow cytometry showed altered cell-cycle distribution (S phase decrease and G0/G1 increase in the siRNA group and siRNA MNPs group compared to the control). **H** Schematic diagram of nude mice inoculated with U87 MG cells and administration. **I**, **J** The tumor volume and average weight were significantly smaller in the siRNA MNPs group than in the control and MNPs groups. **K**, **L** The results of dynamic measurements of tumor volume and mouse body weight in live animals. **M**, **N** EDU labelling revealed a significant decrease in proliferating cells in the siRNA MNPs group compared to the control and MNPs groups. Scale bar is 100 μm in (**D**) and 50 μm in (**M**). Data are represented as mean ± SEM. Two-way repeated measures ANOVA with Bonferroni’s post hoc correction in (**K**, **L**) and one-way ANOVA analysis of variance with Tukey’s multiple comparison in others. *P < 0.05, **P < 0.01, *n* = 3 in (**B**–**G**) and *n* = 8 in (**H**–**N**). (**A**) and (**H**) created with BioRender.

We then asked whether inactivating CELSR2 could be a therapeutic strategy for glioma. To address this hypothesis, we developed magnetic nanoparticles (MNPs) to load *CELSR2*-siRNA. MNPs were doped with zinc (Zn_0.4_Fe_2.6_O_4_) with uniform spherical shape and good dispersion under transmission electron microscope (Supplementary Fig. 4A). MNPs exhibited a high saturation magnetization (92 emu g^−1^), enabling them to be sensitive to magnetic field actuation (Supplementary Fig. 4B). After surface modification (Fig. 7A), the hydrodynamic diameters and zeta potential were measured by dynamic light scattering (DLS): approximately 1516 nm and −27.2 mV in MNPs-DMSA, 483 nm and 40.73 mV in MNPs-PEI) (Supplementary Fig. 4C, D). The presence of PEI not only significantly improved the dispersibility of MNPs through the electrostatic repulsive force of amino groups, but also dramatically changed the surface charges of MNPs. The positive charges on the surface of MNPs-PEI provided the preconditions for electrostatic binding between MNPs-PEI and siRNA. Therefore, we chose MNPs-PEI as carriers of *CELSR2*-siRNA. The biocompatibility of MNPs-PEI was then evaluated in cultured U87 MG cells using a CCK-8 assay. Cultured cells stained by crystal violet staining showed a comparable proliferation ability after incubating with different concentrations of MNPs-PEI (in μg/mL: 0, 12.5, 25, 50, 100, 200) for 72 h (Supplementary Fig. 4E, F), suggesting that MNPs-PEI has good compatibility. To confirm whether *CELSR2*-siRNA could form stable complexes with MNPs-PEI, we performed agarose gel electrophoresis after incubating MNPs-PEI and *CELSR2*-siRNA at mass ratios of 5, 10, 20, 40, and 60. In the gel, the bands of siRNA gradually declined with increasing mass ratios of MNPs-PEI to siRNA and were rarely detectable at the mass ratios of 40 and 60, indicating that they formed stable nanocomplexes (Supplementary Fig. 4G). Therefore, we prepared nanoparticles at a mass ratio of 40 (MNPs-PEI: siRNA) for subsequent experiments.

Cultured U87 MG cells were treated with *CELSR2*-siRNA or MNPs-loaded *CELSR2*-siRNA (Fig. 7A) as the siRNA group and siRNA MNPs group, respectively, and those cells without additional treatment were used as the control. Iron oxide in nanoparticles was visualized by Prussian blue (PB) staining, showing that all U87 MG cells were blue in the siRNA MNPs group (Fig. 7B). Thus, nanoparticles of MNPs-loaded *CELSR2*-siRNA were taken up by glioma cells. We assessed *CELSR2* mRNA levels in cultured U87 MG cells using RT-qPCR and found a significant downregulation in the siRNA group (about 33% of the control) and siRNA MNPs group (about 28% of the control) compared to the control group (Fig. 7C). We found a significant decrease in cell proliferation rate in EDU labelling (Fig. 7D, E) and a cell-cycle distribution (S phage decrease and G0/G1 increase) in flow cytometry (Fig. 7F, G) in the siRNA group and siRNA MNPs group. Thus, *CELSR2*-siRNA and MNPs-loaded *CELSR2*-siRNA similarly knock down CELSR2 expression, decrease proliferation, and disturb cell cycle of cultured glioma cells.

To examine the therapeutic effect of MNPs-loaded *CELSR2*-siRNA on glioma in vivo, we inoculated U87 MG cells subcutaneously in nude mice. When the tumor volume reached 100 mm^3^, mice received intravenous injection of MNPs loaded with and without *CELSR2*-siRNA (siRNA MNPs and MNPs groups respectively) for 7 consecutive days, and a magnet (magnetic field: 0.5 T) was fixed at the inoculation site (Fig. 7H). Mice inoculated U87 MG cells without additional treatment were used as the control. Three weeks later, we collected tumor nodules and found a significant decrease in tumor weight in the siRNA MNPs group compared to the control and MNPs groups (Fig. 7I, J). A similar change was identified in the dynamical measurement of tumor volumes in live animals at different time points (Fig. 7K), while mouse body weight was comparable among the three groups (Fig. 7L). PB stain showed that blue nanoparticles were enriched in tumor tissues in the MNPs and siRNA MNPs groups, but not in the control group (Supplementary Fig. 5A). Immunohistochemical staining revealed a significant downregulation of CELSR2 immunoreactivities in tumor tissues in the siRNA MNPs group compared to the control and MNPs groups (Supplementary Fig. 5B, C). As expected, cell proliferation studied by EDU labelling in tumor tissues showed a significant decrease in the siRNA MNPs group (Fig. 7M, N). Proteins were extracted from tumor nodules and followed by Western blots, showing upregulation of p-β-catenin and GSK-3β and downregulation of CELSR2 and β-catenin in the siRNA MNPs group compared to the control group (Supplementary Fig. 5D, E). These results indicate that intravenous administration of MNPs-loaded *CELSR2*-siRNA effectively inhibits glioma growth in vivo.

## Discussion

Tumorigenesis is a multi-step and complex process, with uncontrolled cell proliferation being a key factor in tumor development and progression. Identifying genes and proteins that promote tumor cell proliferation is critical for developing new methods for cancer diagnosis, treatment, and prognosis. Rapid tumor growth and high recurrence rate after surgical treatment are important reasons for the short survival of glioma patients in clinical practice [11]. Our present study demonstrates that CELSR2 is a potential molecular target for glioma therapy, and inhibiting CELSR2 function may become an important intervention option combined with surgical treatment.

Our previous study has shown that Celsr2 is expressed in intact and reactive mouse astrocytes [25]. In this study, *CELSR2* mRNA was also identified in the normal human astrocyte line (CP-H122), whereas its level was significantly increased in the human glioma cell lines (U87 MG and U251). Immunohistochemical studies of clinical samples confirmed that glioma development was associated with CELSR2 expression elevation. The results are attributed to increasing glial cells in glioma tissues and CELSR2 involvement in the glioma development. The later explanation was confirmed by our study of glioma cell lines (U87) in vitro. After *CELSR2* was knocked down by *CELSR2*-shRNA, proliferation of glioma growth was significantly downregulated as shown by EDU labelling, and cell cycle of growing glioma was arrested as shown by an increase of G0/G1 ratio and a decrease of S phase. *CELSR2* silencing-induced proliferation decrease and cell cycle arrest suggest that CELSR2 negatively regulates expression of proliferation-associated genes. This was confirmed by DIA proteomics analysis of cultured glioma cells. *CELSR2* KD resulted in a significant downregulation of cyclin D1, which is an important regulator of cell cycle and plays a central role in the pathogenesis of cancer [30]. From these findings, we propose that CELSR2 elevation in gliomas (cultured cells and patient samples) is not only because of an increase of proliferating glial cells but also due to CELSR2-regualting proliferation. Thus, CELSR2 is an important biomarker indicating glioma development. In line with this, we found a negative correlation between CELSR2 expression and OS of patients with gliomas. Likewise, it was reported that CELSR2 expression increase was accompanied with short OS in patients with hepatocellular carcinoma [22].

*Celsr* genes (*Celsr*1-3) encode atypical cadherin receptor proteins, which, like Frizzled receptor proteins, belong to core planar cell polarity members and are involved in regulating a variety of biological developmental processes [20]. In neural development, Celsr2 is proposed to steer neuronal migration, cilia development and organization, and axon fasciculation by mediating homophilic interactions between neighboring cells [21, 24, 31]. Because some mutations of Celsr genes have similar developmental abnormalities as mutations of some Frizzled genes, it is also proposed that they share some ligands [20]. It has been well documented that Frizzled members function as the important receptors to Wnts in tumorigenesis via the canonical (β-catenin dependent) and non-canonical (β-catenin independent) pathways [32]. In the Wnt-β-catenin pathway, β-catenin is the key member to control the nuclear transcription process. In the absence of Wnts, β-catenin is phosphorylated by GSK-3β and followed by degradation. Upon Wnts binding to their receptors (like Frizzled4), β-catenin phosphorylation is inhibited and accumulated β-catenin is translocated to nucleus to activate transcription factors in the T-cell factor (TCF)/lymphoid enhancing factor (LEF) family [16].

Our study demonstrates that GSK-3β and β-catenin are the downstream signaling partners of CELSR2 in regulating human glioma growth. Glioma cell culture study showed that *CELSR2* KD induced an upregulation of total GSK-3β (phosphorylated protein decrease) and phosphorylated β-catenin (total protein decrease), and a decrease of TCF/LEF activity, showing an inactive state of the Wnt-β-catenin signaling. In *CELSR2* KD glioma cells, suppressing GSK-3β activation using inhibitors induced downregulation of phosphorylated β-catenin and upregulation of unphosphorylated β-catenin which subsequently activated nuclear transcription and cell proliferation as shown by EdU labeling and cell cycle analysis.

Our findings suggest that CELSR2 acts as one possible receptor of WNT3A ligand to influence human glioma growth. In glioma cell culture, additional administration of WNT5A, WNT3A and WNT1 promoted cell proliferation, which is in line with the clinical report that high expression of WNT5A, WNT3A and WNT1 correlated with short OS of patients with gliomas [27–29]. However, the enhanced glioma proliferation induced by WNT3A, but not by WNT5A or WNT1, was diminished by *CELSR2* silencing. Simultaneously, WNT3A-induced activation of the GSK-3β/β-catenin signaling and enhancement of cell cycle was significantly compromised in cultured *CELSR2* KD glioma cells. Thus, WNT3A enhancing glioma growth is CELSR2-dependent. Taking together, we identify a novel signaling pathway-WNT3A/CELSR2/GSK-3β/β-catenin in the development of gliomas. This proposal is supported by the previous report that silencing CELSR2 inhibited Schwann cell migration and proliferation through the Wnt/β-catenin pathway [23]. Inhibition of Wnt/β-catenin signaling pathway is considered as a key event in tumorigenesis and development [14], and abnormal activation of the Wnt/β-catenin signaling pathway is closely related to the development of gliomas [29]. Combined with our study, CELSR2 is a novel potential therapeutic target for gliomas, and *CELSR2* silencing disrupts the Wnt/β-catenin pathway and induces cell cycle arrest and tumor proliferation inhibition, which is considered as one of the therapeutic strategies in cancer treatment [33]. Future investigations should aim to provide robust experimental evidence supporting the interaction between WNT3A and CELSR2, particularly through co-immunoprecipitation assays. However, several technical challenges must be addressed to facilitate these studies. Notably, the large molecular size of CELSR2 as a transmembrane receptor protein, coupled with the current lack of high-quality antibodies, has consistently posed significant obstacles to the successful execution of such experiments.

We confirm that administration of MNPs-loaded *CELSR2*-siRNA is an effective therapeutic strategy for gliomas. Firstly, subcutaneous inoculation of *CELSR2*-silencing glioma cells in nude mice showed slow tumor growth, decrease of cell proliferation, and inactivation of the Wnt/β-catenin signaling. Secondly, we synthesized MNPs to carry *CELSR2*-siRNA. Our results showed that MNPs maintained good biocompatibility and efficient loading capacity of *CELSR2*-siRNA. Based on the magnetic function of MNPs, intravenously-administered MNPs-loaded *CELSR2*-siRNA could accumulate xenogeneic glioma nodules of nude mice upon a magnet guide. The study in vivo provided strong evidence that administration of MNPs-loaded *CELSR2*-siRNA effectively inhibited cell proliferation and tumor growth.

Our analysis of the clinical sample database demonstrates that CELSR2 expression is significantly upregulated in a substantial subset of glioma specimens. In conjunction with findings from our animal model studies, these data confirm that suppression of CELSR2 expression markedly inhibits glioma growth. Collectively, our studies suggest that CELSR2 represents a promising molecular target for the clinical treatment of glioma. Nevertheless, several challenges must be addressed before its clinical application can be realized. First, further expansion of the clinical sample cohort is required to elucidate the association between CELSR2 expression levels and glioma pathological subtypes, as well as patient prognosis, which is essential for the development of personalized therapeutic strategies. Second, the development of an efficient and safe targeted delivery system for CELSR2-directed gene therapy remains a critical prerequisite for its translation into clinical practice.

In conclusion, our study demonstrates for the first time that CELSR2 promotes human glioma growth via the WNT3A/β-catenin pathway and administration of MNPs-loaded *CELSR2*-siRNA is a promising therapeutic strategy for gliomas.

## Materials and methods

### Mice

Animal experiments were approved by the Laboratory Animal Ethics Committee at the University of Health and Rehabilitation Sciences, China (approval No. 20243002). BALB/C male nude mice (aged 5–6 weeks) were housed in a specific-pathogen-free grade animal room with a room temperature of 25 °C and humidity of 70%, under a 12-h light/dark cycle, with access to water and food. Animal experiments were performed by subcutaneous injection of U87 MG cell suspensions (5 × 10^6^ cells/100 μl) into the left lower back of mice. The experiment was conducted when the tumor volume grew to 100 mm^3^. Tumor volume (calculated as 0.5 × A^2^ × B, where A is the shortest and B is the longest diameter) and the body weight were monitored every 3 days. Following intravenous injection of magnetic nanoparticles (MNPs) at a dose of 25 mg/kg/day, a magnet (magnetic field: 0.5 T) was fixed at the tumor site of the mice for 24 h [34], which was performed for 7 consecutive days. At the end of treatment, mice tumors were collected, photographed, and weighed. The treatment protocols for the in situ glioma tumor models are described in detail in the Supplementary Materials. In this study, animals were assigned to different experimental groups using a computer-generated sequence of random numbers. This study used blinding to reduce bias. Researchers were unaware of the group allocation during the experiment and when evaluating the results, ensuring blinding and reducing potential bias.

### Clinical samples

The study was approved by the Clinical Research Ethics Committee of the First Affiliated Hospital of Jinan University School of Medicine, China (approval No.KY-2024-110), and complied with the guidelines of the Declaration of Helsinki. Signed informed consents were obtained from all participants prior to specimen collection. Fresh glioma tissue were collected during resection at the First Affiliated Hospital of Jinan University. All human tissues were frozen in liquid nitrogen within 30 min of surgical removal and stored at −80 °C. The selected samples were then subjected to detection of CELSR2 expression using reverse transcription quantitative polymerase chain reaction (RT-qPCR), Western blots or immunohistochemical staining.

### Cell culture

U87 MG, U251 cell lines and Human astrocytes CP-H122 were purchased from Pricella (Wuhan, China). All cells were maintained in standard tissue culture incubators at 37 °C and 5% CO_2_. Cells were cultured in DMEM-F12 supplemented with 10% fetal bovine serum (FBS, Gibco, 10099-141). The isolation and culture procedures for primary glioma cells are described in detail in the Supplementary Materials. To estimate the effects of CELSR2 inactivation in U87 MG, lentivirus (2 × 10^9^ TU/ml) encoding *CELSR2*-shRNA, or *CELSR2*-siRNA was added to the culture medium, and fresh culture medium was added 24 h later. The knockdown efficiency of *CELSR2* was detected by RT-qPCR or Western blots. *CELSR2* shRNA and siRNA primer sequences are provided in Supplementary Table 1. The primer sets with the highest knockdown efficiency were selected for subsequent experimental procedures (shCELSR2 #1 and siCELSR2 #2, Supplementary Fig. 6). The drugs used in the study were as follows: TWS119 (3 μM, 24 h, MCE, HY-10590), WNT5A (100 ng/ml, 24 h, Abcam, ab204627), WNT3A (100 ng/ml, 24 h, MCE, HY-P70453A), WNT1 (100 ng/ml, 24 h, Abcam, ab84080). Cells were transfected with TOPFlash plasmids (Beyotime, D2501) or FOPFlash plasmids (Beyotime, D2503) for 24 h, and the reporter genes were detected with the luciferase reporter enzyme gene detection kit (Beyotime, RG005).

### Cell proliferation assay

The CCK-8 test, EdU labeling and colony formation assay were used to assess cell proliferation. The detailed protocols were described in the Supplementary Materials.

### RT-qPCR

Total RNA of cells was extracted using the TriZol Reagent (Invitrogen, Carlsbad, CA, USA) according to the manufacturer. cDNA was synthesized from total 1 μg RNA using the Reverse Transcription System (Promega) and 1 μl cDNA subjected to PCR using the Eco™ Real-Time PCR System (Illumina). Primers were listed in Supplementary Table 2. The expression level was evaluated using the 2^−ΔΔCt^ method.

### Histopathology and immunohistology staining

Mice were sacrificed and partial tumor tissue were fixed in 10% formalin, embedded in paraffin and sections (3–4 µm) for immunohistochemistry or Prussian blue staining (Prussian Blue Stain Kit, Abcam, ab150674). The sections were incubated with antibody against CELSR2 (1:200, NOVUS, NLS1943) overnight at 4 °C, followed by incubation with the secondary antibody for 1 h.

### Western blots

Protein extracts from tumor samples, or cultured U87 were analyzed on 10% sodium dodecyl sulfate polyacrylamide gels and transferred to 0.45 μm polyvinylidene fluoride membranes. The antibody information is detailed in the Supplementary Materials. Immunoreactivity was detected using an enhanced chemiluminescence detection kit (Bio-Rad, 1705061), images were captured using the ChemiDoc™ Touch Imaging System (Vilber, France), and signals were quantified using ImageJ. The original images of the Western blot strips can be found in the Supplementary Materials-Western blots.

### Flow cytometry analysis

Flow cytometry was used to monitor cell apoptosis and cycle distribution by Apoptosis and Cell Cycle Analysis Kit (Invitrogen, V13242). Briefly, 1 × 10^6^ Cells were collected and added Annexin V-FITC and PI to detect apoptosis, or only added PI after cells fixed with 70% ethanol to detect cell cycle by flow cytometry.

### Synthesis of PEI modified magnetic nanoparticles (MNPs) and siRNA loading to MNPs

MNPs were prepared based on the previous work [35]. The detailed synthesis protocols and characterization of MNPs were described in the Supplementary Materials. *CELSR2*-siRNA was dissolved in DEPC water and then added to MNPs. The mixture was followed by vertexing the solution for 10 seconds and incubating for 30 min at room temperature.

### Data-independent acquisition (DIA) proteome analysis

Total protein was isolated from U87 MG cells with or without *CELSR2* knockdown using Radio Immunoprecipitation Assay Lysis buffer (RIPA). Protein sequencing and protein libraries construction were performed by the Guangzhou Jidiao and data analysis was conducted by OmicShare and Omicsmart. Criteria for differential protein expression included coefficient of variation <0.5, average ratio-fold change ≥1.5 or ≤0.67, and *P* value < 0.05 in the Student’s *t*-test between groups.

### Statistical analysis

Results are presented as mean ± SEM. Statistics were performed using GraphPad Prism 7.04. Data were analyzed using Student’s *t*-test, one-way ANOVA analysis of variance with Tukey’s multiple comparison tests or Two-way repeated measures ANOVA with Bonferroni’s post hoc correction (Prism®, 7.0c, GraphPad, San Diego, CA, USA). The significance level was set as *P* < 0.05. *P < 0.05; **P < 0.01; ***P < 0.001; and ****P < 0.0001.

## Supplementary information

Supplementary Materials

Supplementary Materials-Western blots

## Data Availability

The data generated by this study are available in the article and its Supplementary data files. All additional data supporting the findings of this study are available upon reasonable request by the corresponding author. The DIA sequencing data are available via ProteomeXchange with identifier PXD060044.
