# Supplementary material for: Atypical cadherin CELSR2 acts as a therapeutic target for glioma through WNT3A/β-catenin signaling
Source: Cell Death Dis. 2025 Nov 3;16(1):786. doi: 10.1038/s41419-025-08116-8 (PMC12583761; doi:10.1038/s41419-025-08116-8)
Supplement: Supplementary file 1 — Supplementary Materials [file 41419_2025_8116_MOESM1_ESM.doc]

**Supplementary Materials**

**Title: Atypical cadherin CELSR2 acts as a therapeutic target for glioma through WNT3A/β-catenin signaling**

**Materials and methods**

**Cell proliferation assay**

**CCK-8**: Cells (1 × 10^3) were seeded in 96-well plates for 1, 2, 3, 4 and 5 days. Cell proliferation activity was measured using Cell Counting Kit-8 (CCK8, Dojindo Molecular Technologies, CK04-11) according to the manufacturer's protocol.

**EdU labeling:** Cells (1 × 10^5 cells/ml) were seeded on 18-mm coverslips. After 24 hours of incubation with medium, cells were treated by 10 μM EdU (EdU Cell Proliferation Kit with Alexa Fluor 555, Beyotime, C0075S) for 24 hours. They were then fixed with 4% paraformaldehyde for 15 minutes, washed with phosphate-buffered saline (PBS) containing 3% bovine serum albumin (BSA), and incubated with the Click Additive Buffer for 15 min. Hoechst 33342 was used to stain nuclei.

**Colony formation assay**

Cells (5×10^2) were plated in six-well plates and grown for 14 days with the indicated treatment. Then, cells were fixed with 4% paraformaldehyde for 15 minutes and stained with 0.05% crystal violet for more than 1 hour. Colonies were counted with a dissecting microscope with which diameters larger than 200 μm were counted.

**Synthesis of PEI modified magnetic nanoparticles**

Firstly, 2.00 mmol of iron (III) acetylacetonate and 1.3 mmol of zinc chloride were placed in a 100 ml three-neck round-bottom flask containing 12.62 mmol oleic acid, 24.32 mmol oleylamine, and 13.72 mmol octylether. The mixture was then heated to 330 ºC for 1 hour under an argon atmosphere. After cooling down to room temperature, ethanol was added to the mixture, leading to a black precipitate of MNPs. Next, the MNPs were washed with ethanol three times and isolated using a magnetic field. Finally, the MNPs was dispersed in toluene and stored at 4 ℃ for next step. To further improve the dispersion and biocompatibility, MNPs were transformed from oil phase to water phase via surface double-exchange. Briefly, 25 mg of dimercaptosuccinic acid (DMSA) was dissolved into 5 mL of dimethyl sulfoxide (DMSO), and 5 mg of MNPs were dispersed into 10 ml ethanol. The ethanol solution of MNPs was then sonicated using an ultrasonic probe at the 50% amplitude for 90 minutes, and DMSA solution was added dropwise. The functional groups of COOH in DMSA have a strong tendency to coordinate with metals and strong hydrophilicity. After modification, the product (MNPs-DMSA) was washed three times with deionized water and collected by magnetic separation. To endow MNPs with the ability to load siRNA, polyethyleneimine (PEI, Mw=10,000) was introduced. Firstly, 5 mg of MNPs-DMSA were dispersed into 6 ml deionized water, and 0.6 mmol N-hydroxysuccinimide (NHS) and 0.5 mmol 1-(3-dimethylaminopropyl)-3-ethyl carbodiimide hydrochloride (EDC) were added to activate the carboxyl groups of MNPs-DMSA for reacting with the amino groups of PEI (MNPs-PEI). After an overnight reaction, MNPs-PEI were washed with deionized water three times and stored at 4 ℃.

**Characterization of MNPs**

The morphology of MNPs was charactered using transmission electron microscopy (TEM, JEM-1230, JEOL Ltd.). The particle size was statistically analyzed using the software of Nano Measurer 1.2. The zeta potential and hydrodynamic size of MNPs-DMSA and MNPs-PEI were analyzed by dynamic light scattering (DLS, Mastersizer 2000, Malvern, Worcestershire, UK). The static magnetic properties of dry MNPs were measured using a Vibrating Sample Magnetometer (VSM, Lakeshore 7407, US). MNPs-PEI prepared with different mass ratios (MNPs: siRNA) were subjected to 3% agarose gel electrophoresis at 90 mV for 30 min, after which the gels were stained with ethidium bromide solution and observed under a GelDoc imaging system (Bio-Rad, CA, USA).

**The antibodies of Western blots**

The primary antibodies were used: anti-CELSR2 (1:500, NOVUS, NLS1943), anti-β-catenin (1:1000, cell signaling technology, #9562), anti-phospho-β-Catenin (Ser552) (1:1000, cell signaling technology, #9566), anti-GSK-3β (1:1000, Beyotime Biotechnology, AG751), Anti-phospho-GSK-3β (1:1000, Beyotime Biotechnology, AF1531), anti-cyclinD1 (1:1000, cell signaling technology, #55506), anti-β-tubulin rabbit polyclonal antibody (1:1000; Abcam, ab18207), and anti-GAPDH mouse polyclonal antibody (1:1000; Abcam, ab8245); the secondary antibodies were peroxidase anti-rabbit IgG (1:10000; Abcam, ab6721) and peroxidase anti-mouse IgG (1:10000; Abcam, ab6789).

**Isolation and Extraction of Primary Glioma Cells**

During the operation, glioma tissue were removed and immediately placed in pre-cooled phosphate-buffered saline (PBS). Approximately 2-5 ml of tumor core tissue was carefully extracted from the specimens for primary glioma cell isolation. The glioma tissue was then cut into small pieces, each measuring 1 mm³. Any attached membrane structures and blood vessels were meticulously removed, and the glioma tissue was further minced into smaller fragments. Next, 0.25% trypsin was added to the minced glioma tissue at a volume ratio of 1:1, ensuring thorough mixing of the mixture. The mixture was then transferred to a cell culture incubator for digestion for approximately 1 hour. After digestion, the primary cell complete culture medium (DMEM-F12 supplemented with 20% fetal bovine serum and 1% Penicillin-Streptomycin Solution) was added to the mixture at a volume ratio of 1:1. The resulting mixture was filtered through a 40 μm sieve and then centrifuged at 300 g, 5 minutes. The primary glioma cell was resuspended in complete culture medium. And were maintained in standard cell culture incubators at 37°C and 5% CO_2_.

**Treatment of glioma in an** **orthotopic model**

In the animal experiment involving the glioma orthotopic tumor model, a U87 MG cell suspension (1×10^5 cells/μL, 2μL) was injected into the striatum via intracranial injection (ML: +2; AP: +1; DV: +3). Seven days after a U87 MG cell injection, in vivo bioluminescence imaging was conducted to determine whether a tumor had formed. Subsequently, lentivirus injection (LV-shCELSR2: 3x10^9^ TU/Ml, 1μL) was performed at the tumor site(ML: +2; AP: +1; DV: +3). One month after the cell injection, in vivo bioluminescence imaging was performed again to monitor the growth of the glioma. The body weight of the mice was monitored every 3 days throughout the experiment. At the end of the treatment, the mice brains were collected, weighed, and stained for further analysis.

**Bioluminescent reporter imaging**

The glioma was monitored at 7 days and 28 days after U87 MG cells injection by bioluminescent reporter imaging. In more detail, intraperitoneal injection of D-Luciferin (absin, Cat# abs42075819) DPBS solution at a 150 mg/kg dosage at the indicated time. The *in vivo* fluorescence imaging was performed using the BLT AniView Pro system (AniView600 Pro, BLT, Guangzhou, China) after 15 min of D-Luciferin injection. The fluorescence intensities were analyzed with AniView Pro Capture software within the same gray scale range.

**Supplementary Table 1. Primers for shRNA and siRNA**

| **Genes** | **Forward Sequence** | **Reverse Sequence** |
| --- | --- | --- |
| *ShCelsr2 #1* | CCGGACTCGTCAGGCTCCGAATTTCCTCGAGGAAATTCGGAGCCTGACGAGTTTTTTG | AATTCAAAAAACTCGTCAGGCTCCGAATTTCCTCGAGGAAATTCGGAGCCTGACGAGT |
| *ShCelsr2 #2* | CCGGCGCTTGGACAAAGGGAACTTTCTCGAGAAAGTTCCCTTTGTCCAAGCGTTTTTG | AATTCAAAAACGCTTGGACAAAGGGAACTTTCTCGAGAAAGTTCCCTTTGTCCAAGCG |
| *ShCelsr2 #3* | CCGGCCACTATACAGTGAATGTTAACTCGAGTTAACATTCACTGTATAGTGGTTTTTG | AATTCAAAAACCACTATACAGTGAATGTTAACTCGAGTTAACATTCACTGTATAGTGG |
| *si Celsr2 #1* | ACUCGUCAGGCUCCGAAUUUCdTdT | GAAAUUCGGAGCCUGACGAGUdTdT |
| *si Celsr2 #2* | CCACUAUACAGUGAAUGUUAAdTdT | UUAACAUUCACUGUAUAGUGGdTdT |
| *si Celsr2 #3* | CCAUUCUGUCCUUCGAUUAUGdTdT | CAUAAUCGAAGGACAGAAUGGdTdT |
| *si Celsr2 #4* | CGCUUGGACAAAGGGAACUUUdTdT | AAAGUUCCCUUUGUCCAAGCGdTdT |

**Supplementary Table 2. Primers for RT-qPCR**

| **Genes** | **Forward Sequence** | **Reverse Sequence** |
| --- | --- | --- |
| Celsr2 | AGTGGCATACGGTGCAGCTGAA | ACTCCTGTGTCACAGCCATCCA |
| β-catenin | CACAAGCAGAGTGCTGAAGGTG | GATTCCTGAGAGTCCAAAGACAG |
| Cyclin D1 | TCTACACCGACAACTCCATCCG | TCTGGCATTTTGGAGAGGAAGTG |
| GSK-3β | CCGACTAACACCACTGGAAGCT | AGGATGGTAGCCAGAGGTGGAT |
| *GAPDH* | CCAATGTGTCCGTCGTGGATCT | GTTGAAGTCGCAGGAGACAACC |


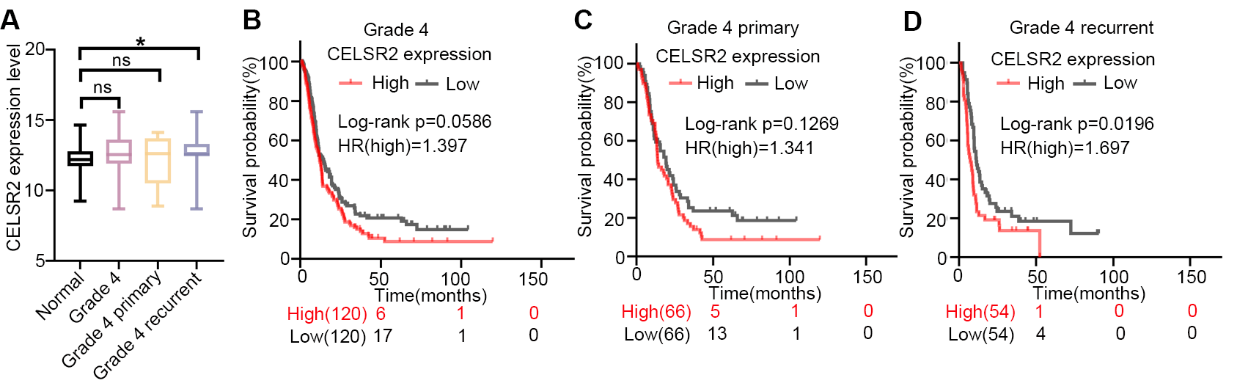


**Supplementary Fig. 1 CELSR2 expression and effect on overall survival in patients with recurrent glioma of grade 4.**

(A) Analysis of TCGA data reveals significantly elevated *CELSR2* mRNA levels in recurrent glioma tissues of Grade 4 compared to normal tissues (n=1081 for normal; n=248 for Grade 4; n=138 for primary glioma tissues of Grade 4; n=110 for recurrent glioma tissues of Grade 4). (B-D) Kaplan–Meier survival curves illustrate the overall survival in patients with glioma of Grade 4, primary glioma tissues of Grade 4 and recurrent glioma tissues of Grade 4. n=120 in B, n=66 in C, n=54 in D. **P* < 0.05, one-way ANOVA analysis of variance with Tukey's multiple comparison in A.


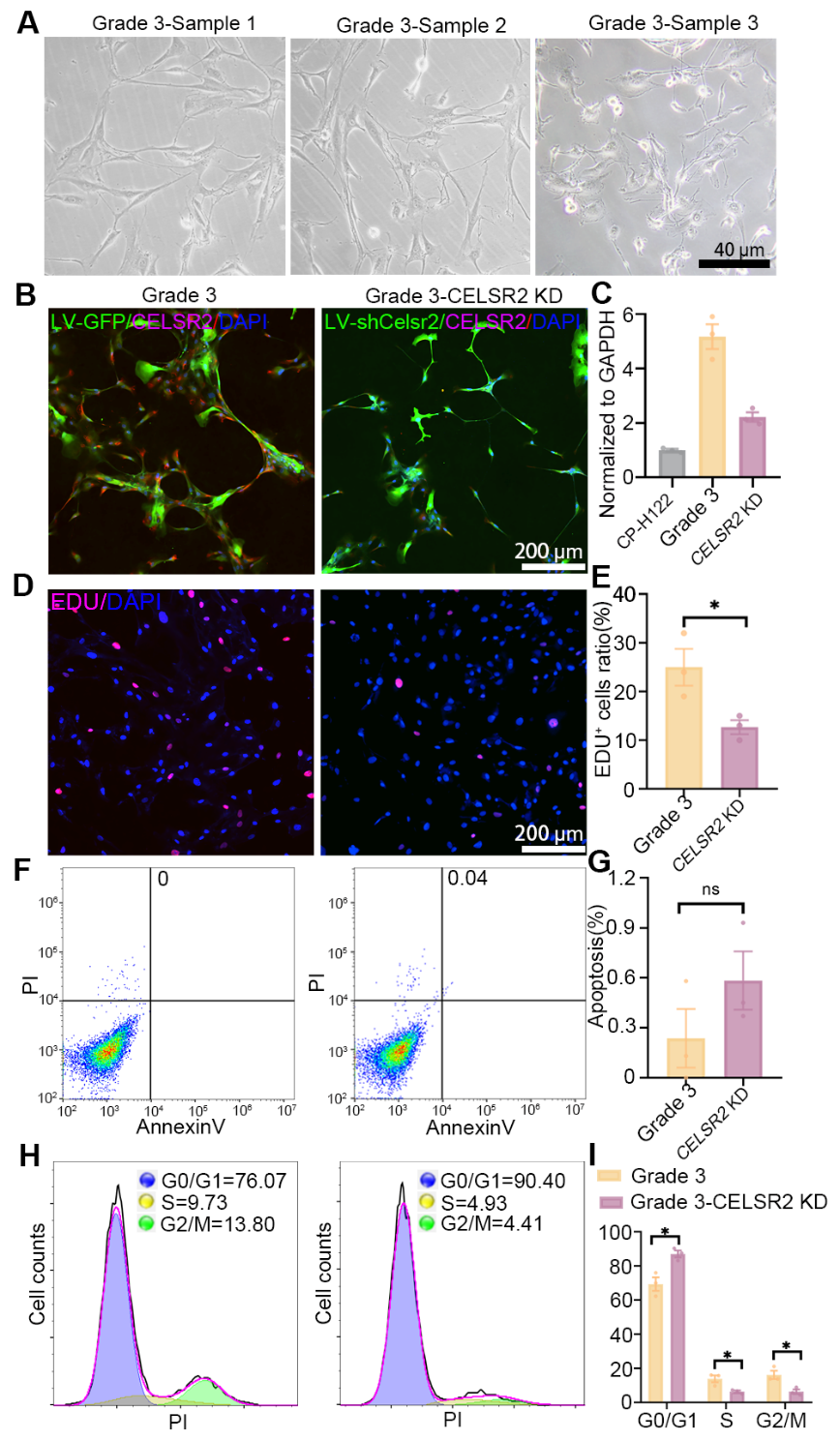


**Supplementary Fig. 2 *CELSR2* KD inhibits primary astroglioma cells proliferation and disturbs cell cycle.**

(A) The cell morphology of primary astroglioma in 3 patients with grade 3 astrocytoma (B,C) Anti-CELSR2 immunofluorescence staining showed the expression of CELSR2 in primary astroglioma cells transfected with the vector (pLVX-shRNA-ZsGreen-puro, control) or *CELSR2*-shRNA (*CELSR2* KD), and the levels of *CELSR2* mRNA were further assessed by RT-qPCR, showing a downregulation in primary astroglioma cells transfected with *CELSR2*-shRNA. (D, E) EDU staining showed a significant decrease in the number of proliferating cells in the CELSR2-KD group. (F, G) Using annexin V and propidium iodide (PI) staining, FACS analysis identified a comparable percentage of apoptotic cells in the CELSR2-KD group and the control group. (H, I) FACS analysis of PI-stained cells showed a significant increase in the G0/G1 ratio and a significant decrease in the S and G2/M phase in the CELSR2-KD group. Scale bar is 40 μm in A and 200 μm in B, D. Data are represented as mean ± SEM. *P < 0.05, Student's t-test, n = 3.

**
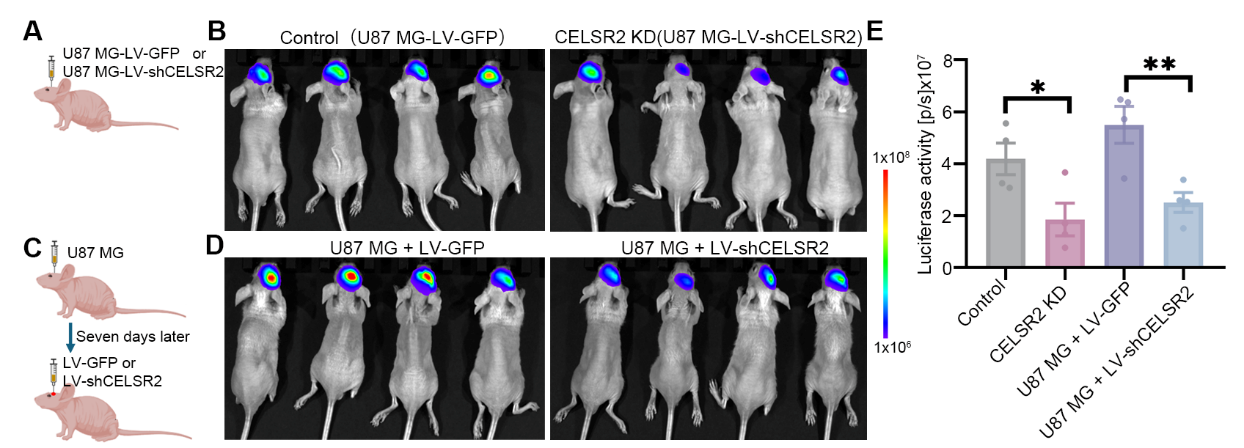
**

**Supplementary Fig. 3 Glioma growth is suppressed by LV-shCELSR2 administration in an orthotopic glioma model.**

(A) Schematic illustration of U87 MG-Luc or U87 MG-CELSR2 KD-Luc cell implantation in BALB/c-nu mice. (B) In vivo bioluminescence imaging results one month after cell implantation. (C) Schematic illustration of U87 MG-Luc cell implantation followed by LV-shCELSR2 injection in BALB/c-nu mice. (D) *In vivo* bioluminescence imaging results one month after LV-shCELSR2 administration. (E) Quantitative analysis of brain photon flux in each experimental group.

Student's *t*-test, **P* < 0.05, ***P* < 0.01, n =4.


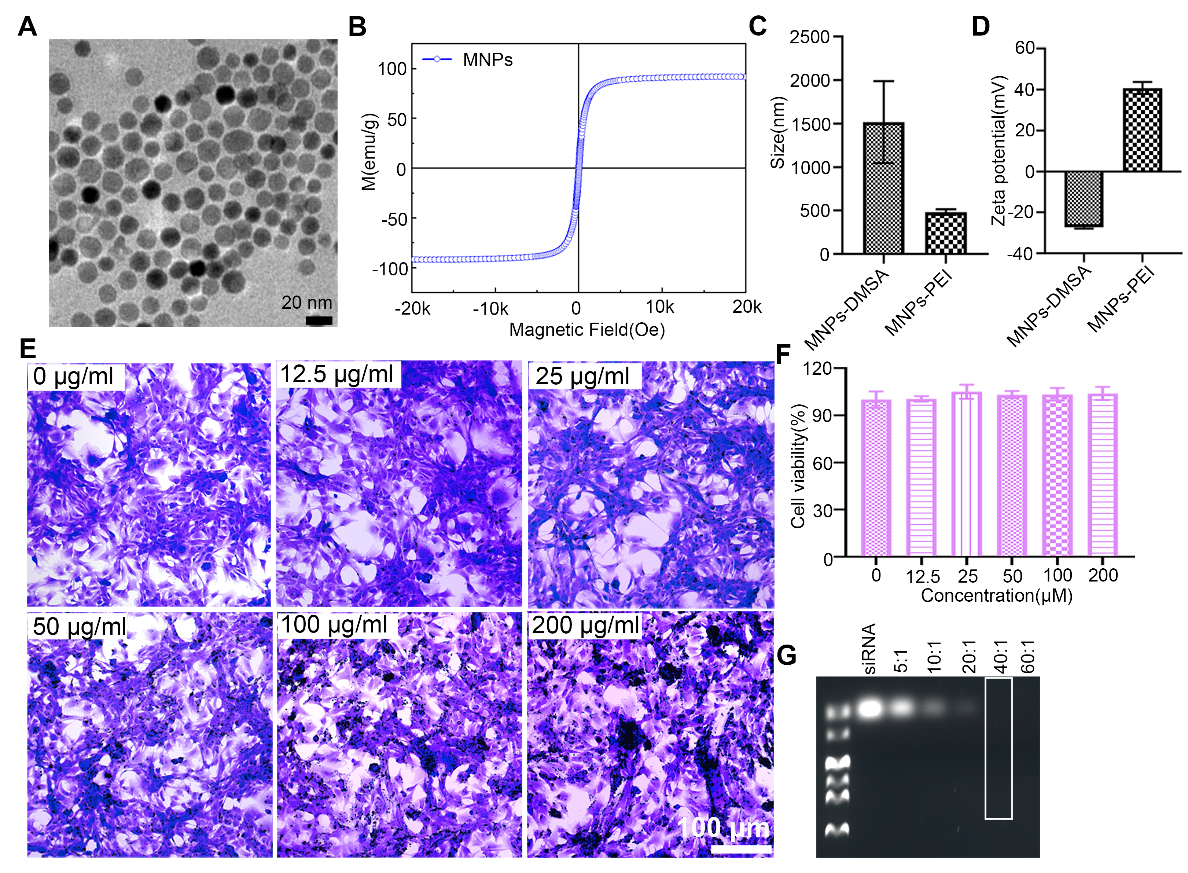


**Supplementary Fig. 4 Characterization and biocompatibility of MNPs**.

(A) Observation of MNPs morphology under TEM revealed uniform spherical shape and good dispersion. (B)The curve displayed the magnetization of dry MNPs in the magnetic field. (C, D) The size and zeta potential of MNPs-DMSA and MNPs-PEI. (E, F) Cell viability and microscope images of cultured U87 MG cells incubated with different concentration of MNPs-PEI for 72 hours showed no significant toxicity. (G)Agarose gel electrophoresis showed that the bands of isolated siRNA gradually declined following increase in mass ratios of MNPs-PEI to siRNA and were rarely detectable at the mass ratios of 40 and 60. Scale bar is 20nm in A and 100 μm in E. Data are represented as mean ± SEM. n=5 in E, F.


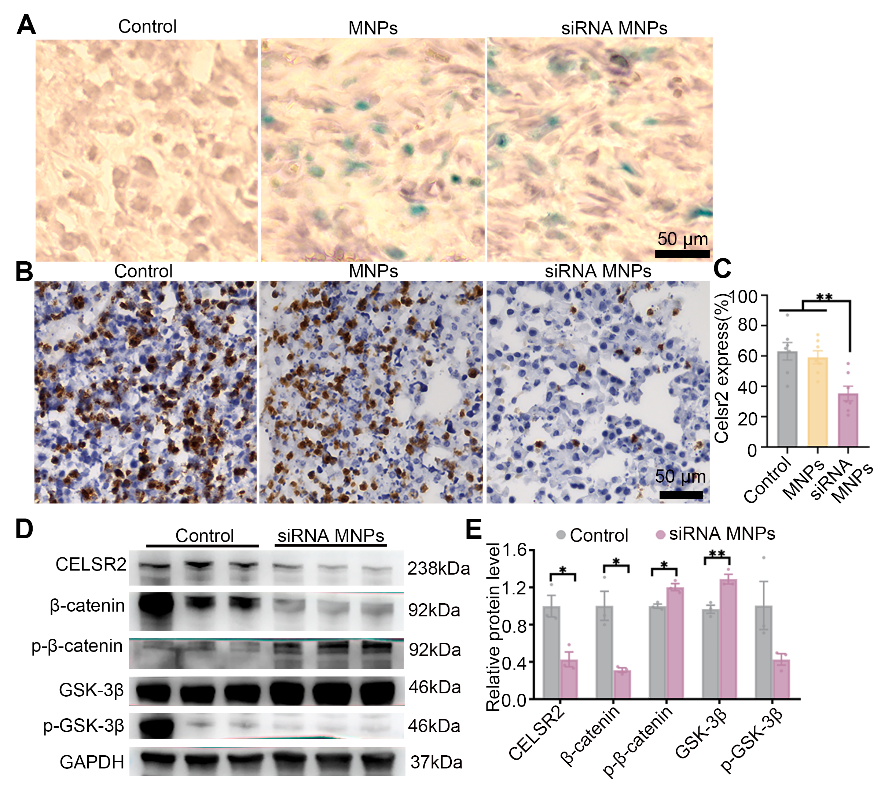


**Supplementary Fig. 5 Administration of MNPs-loaded *CELSR2*-siRNA inhibits CELSR2 expression and function *in vivo***.

1. Prussian blue staining showed that iron oxide-containing blue nanoparticles were scattered in the tumor tissue in the MNPs and siRNA MNPs group, but not in the control group. (B, C) Immunohistochemical staining showed that CELSR2 expression was significantly decreased in the tumor tissue in the siRNA MNPs group compared to the control and MNPs groups. (D, E) Western blots revealed the upregulation of p-β-catenin and GSK-3β and the downregulation of CELSR2 and β-catenin in the tumor nodule in the siRNA MNPs group compared to the control. Data are represented as mean ± SEM. Scale bar is 50 μm. **P* < 0.05, ***P* < 0.01, A-C: n = 7, D-E: n = 3, one-way ANOVA analysis of variance with Tukey's multiple comparison in C, Student's *t*-test in E.


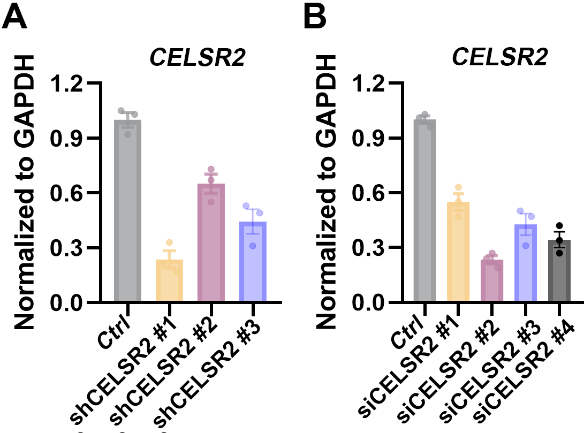


**Supplementary Fig. 6 The knockdown efficiency of *CELSR2* in U87 MG cells by LV-shCELSR2 and CELSR2 siRNA.**

(A) The knockdown efficiency of *CELSR2* in U87 MG cells by LV-shCELSR2. n=3

(B) The knockdown efficiency of *CELSR2* in U87 MG cells by CELSR2 siRNA. n=3
