# Supplementary figures and images for: Atypical cadherin CELSR2 acts as a therapeutic target for glioma through WNT3A/β-catenin signaling
Source: Cell Death Dis. 2025 Nov 3;16(1):786. doi: 10.1038/s41419-025-08116-8 (PMC12583761; doi:10.1038/s41419-025-08116-8)

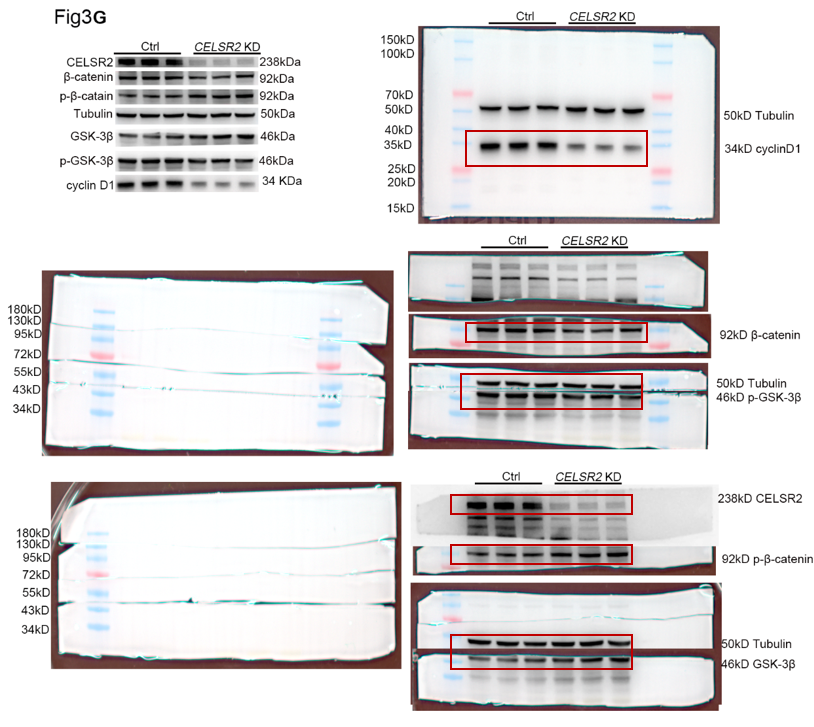


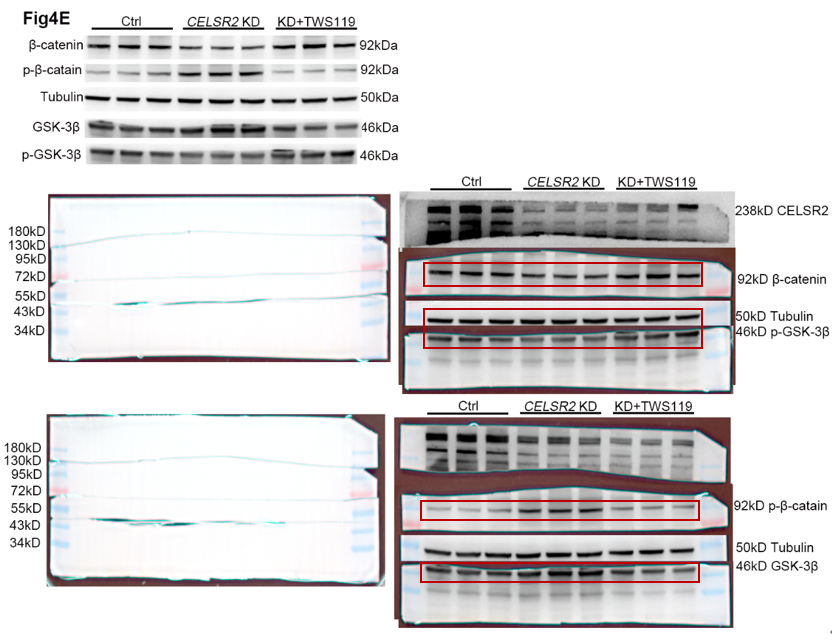


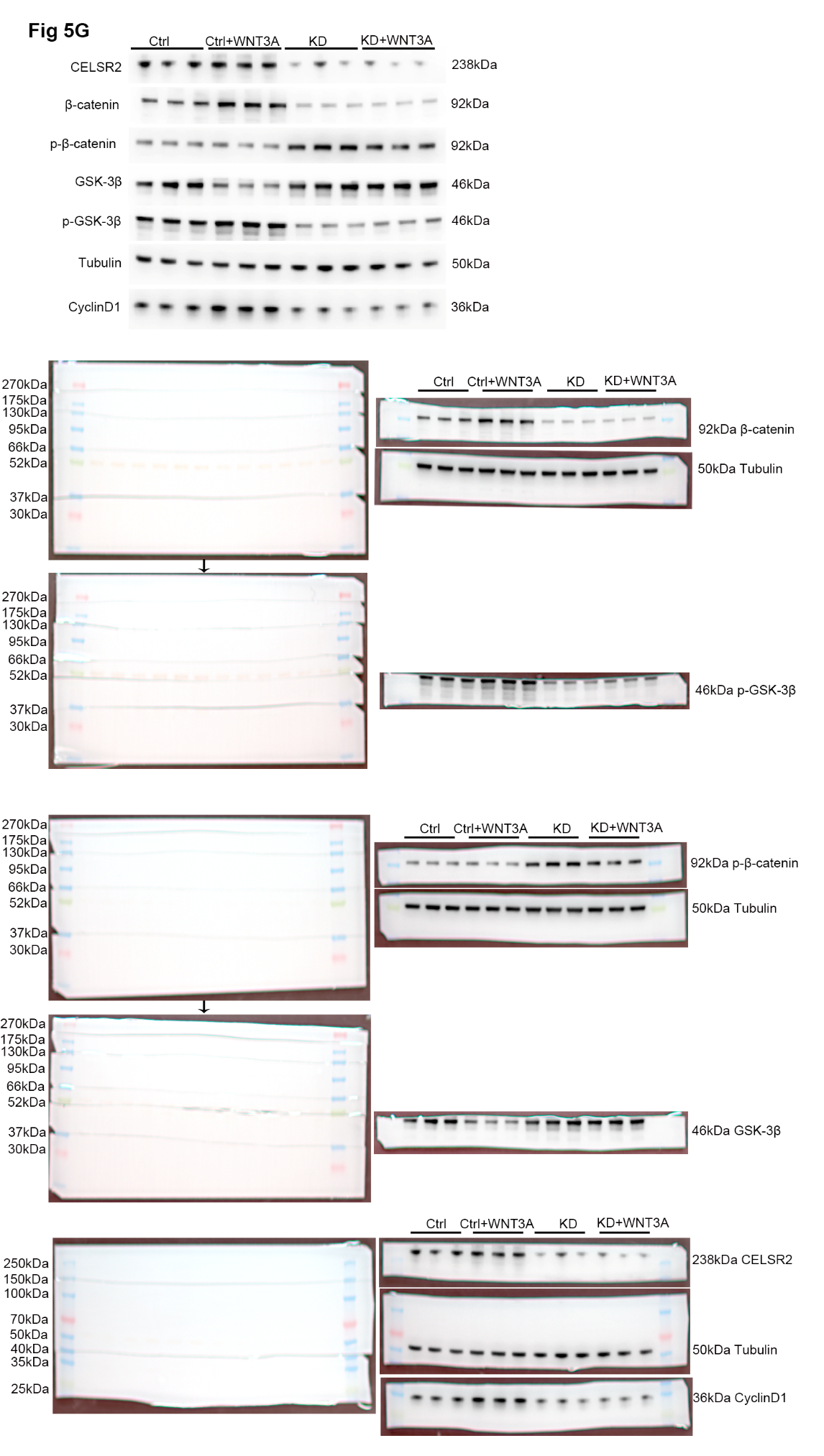


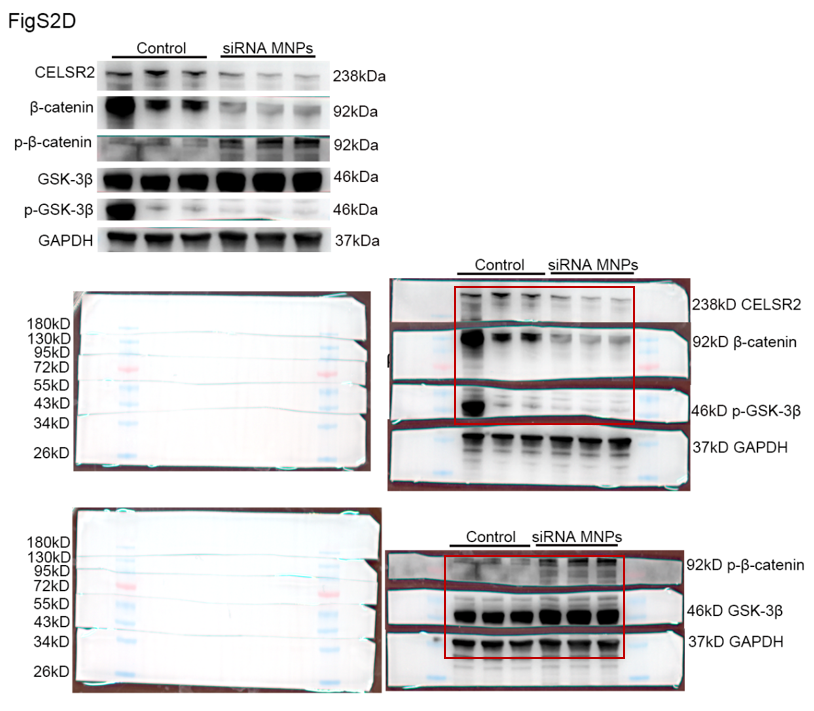

Supplement: Supplementary file 2 — Supplementary Materials-Western blots [file 41419_2025_8116_MOESM2_ESM.docx]
